# Periodic mRNA synthesis and degradation co‐operate during cell cycle gene expression

**DOI:** 10.1002/msb.20140001

**Published:** 2014-04-25

**Authors:** Philipp Eser, Carina Demel, Kerstin C Maier, Björn Schwalb, Nicole Pirkl, Dietmar E Martin, Patrick Cramer, Achim Tresch

In this Addendum, we provide Supplementary Table S6 which contains the periodicity scores of all genes as calculated by the MoPS algorithm. Supplementary Table S6 further contains the cell‐cycle averaged expression values for the labeled and the total mRNA fraction of each gene, together with a binary flag indicating the top 479 genes that were called periodic by MoPS. For the periodic genes, the Table additionally provides the estimated period length, amplitude, phase, and synchrony decay parameters.

## Supplementary Material

Supplementary Table S6

