# Supplementary material for: Periodic mRNA synthesis and degradation co‐operate during cell cycle gene expression
Source: Mol Syst Biol. 2014 Jan 31;10(1):717. doi: 10.1002/msb.134886 (PMC4023403; doi:10.1002/msb.134886)
Supplement: Supplementary file 1 — Supplementary Information and Supplementary Figures S1–S38 [file MSB-10-1-717-s1.pdf]

Contents

1 Model-based screening algorithm for the identification of periodic fluctuations in time series (MoPS) 2

1.1 Description of the overall strategy 2

1.2 Preprocessing and Error Model 2

1.3 Definition of periodic and non-periodic test functions 4

1.4 Kernel representation of the estimation problem. 5

1.5 Parametrization of and screening for periodic genes 7

2 Application of MoPS to cell cycle cDTA data 11

2.1 Data exploration and quality control 11

2.2 High precision estimates of mRNA synthesis rates and half lifes 11

2.3 Significance of MoPS periodicity scores 12

2.4 Estimation of the global parameters cell-cycle length and variation 17

2.5 Estimation of gene-specific parameters 17

2.6 Quantification of absolute mRNA abundance 20

2.7 Cyclins and histones peak timing define cell-cycle stages 20

2.8 mRNA synthesis of non-periodic genes during the cell cycle 25

2.9 Validation of MoPS 25

2.9.1 Comparison with other cell-cycle expression studies 25

2.9.2 Benchmark on identification of bona-fide cell-cycle genes 25

2.9.3 Robustness of peak time assignment 27

2.10 Regulation of periodic mRNA synthesis timing by TFs 28

3 Dynamic RNA turnover model and screen for periodic fluctuations in RNA degradation 31

3.1 A model for mRNA synthesis and degradation 31

3.2 Model specification 32

3.3 Detection of genes with variable degradation rate 32

3.4 Sensitivity and specificity of the Variable Degradation Score in simulated data 33

3.5 The time shift of degradation vs synthesis determines the efficiency of regulation 34

4 Modeling of mRNA synthesis and degradation in cDTA cell cycle data 36

4.1 Improvement of variable degradation model over constant degradation model 36

4.2 Transcription regulation by degradation rate peaks subsequent to synthesis rate peaks . 36

4.3 Correlation between the periodicity score and the variable degradation score 40

5 Supplementary Tables **42**

References **44**

1 Model-based screening algorithm for the identification of periodic fluctuations in time series (MoPS)

1.1 Description of the overall strategy

The MoPS algorithm is designed to recognize periodic behavior in a observation time series $g = (g(t_1), g(t_2), ..., g(t_K))$, having in mind the application to gene expression time series in our cell cycle data. We will use a likelihood ratio statistic to decide whether a time series displays periodic fluctuations or not. To that end, we will define a family of test functions $\mathcal{F}$, which consists of functions that we believe to exhaustively represent time courses of periodically expressed genes. On the other hand, we will define a set of non-periodic test functions, $\overline{\mathcal{F}}$, that we believe to represent all typical time courses of genes that are not periodic, e.g. constant genes, or genes that show temporal drift (monotonically increasing/decreasing genes). Given a time course measurement g, and a continuous function f, let $L(f;\, g)$ denote the likelihood of f, given the observations on g. We determine the maximum likelihood fit $f_g \in \mathcal{F}$ respectively $\overline{f}_g \in \overline{\mathcal{F}}$ for the likelihood function specified in Section 1.2. Our test statistic, termed periodicity score, becomes

$$log \frac{L(f_g;\, g)}{L(\overline{f}_g;\, g)} \qquad (1)$$

The larger the periodicity score, the more likely g shows periodic fluctuations.

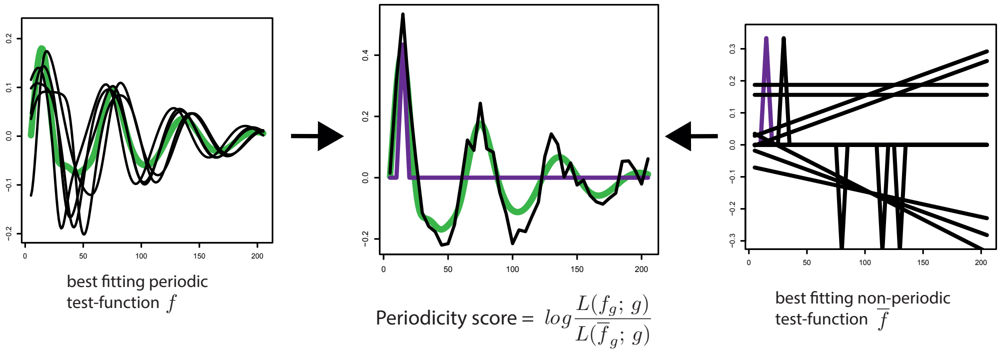

Figure 1: MoPS periodic and non-periodic test-functions. Illustration of the statistical test used in MoPS to determine periodicity in time series data.

1.2 Preprocessing and Error Model

The raw total and labeled mRNA level measurements were corrected for 4-thiouridine labeling bias as described in [9]. The cDTA protocol uses spike-in control RNAs of *S.pombe* as an internal standard to normalize total mRNA arrays (resp. labeled mRNA arrays) between time points. We multiplicatively rescaled all total measurements such that the sum of all total gene expression levels at time zero equals

$6 \cdot 10^4$, a recent estimate of the number of transcripts per *S.cerevisiae* cell [13]. Be aware that all results in this manuscript do not depend on this choice, the rescaling merely facilitates the interpretation of the data in terms of absolute transcript numbers. The true ratio between the (mean) labeled expression measurements and the (mean) total expression measurements of all genes cannot be obtained from our measurements. This normalization factor was derived from the mean transcript half life in *S.cerevisiae* in wild type conditions, and it was chosen as in [12].

It has been pointed out by [2] that erratic deviations in lowly abundant genes (whose measurements have a high coefficient of variation) might cause good periodic fits and hence false periodic gene calls if one assume constant errors (constant variance of measeurements) across the whole range of gene expression. We account for this by using a heteroscedastic error model. Let $g(t_k, i)$ denote the (normalized) measurement of gene g, $g \in G$, at time t_k, $k = 1, .., K$, in replicate $i \in I$. Let $g = (g(t_k, i);\, k = 1, ..., K,\, i \in I)$. The likelihood function $L(f;\, g)$ measures the goodness-of-fit by which a continuous function f approximates g at the measurement time points $t_1, ..., t_K$. Our likelihood function itself is standard, we assume independence of observations, and, as usual for gene expression measurements, Gaussian errors on the logarithmic values of g,

$$L(f;\, g) = \prod_{i \in I} \prod_{k=1}^{K} \frac{1}{\sqrt{2\pi}\sigma_{g,t_k}} \exp\left(-\frac{(\log g(t_k, i) - \log f(t_k))^2}{2\sigma^2_{g,t_k}}\right) \tag{2}$$

For each gene $g \in G$, we measured each time course (labeld or total RNA) in two replicates, namely $g = (g(t_k, i))$. Denote by Θ_g the full parameter set (specified in Section 3.2) which characterizes the approximation functions for g. Our target function is the negative log likelihood $l(\Theta_g;\, g)$,

$$l(\Theta_g;\, g) = \sum_{i \in I} \sum_{k=1}^{K} \frac{(\log\ g(t_k, i) - \log\ \hat{g}(t_k; \Theta_g))^2}{2 \cdot \sigma^2_{g,t_k}} \tag{3}$$

where $\hat{g}(t_k; \Theta_g)$ is the approximation function for g. Our loss function combines the idea of measuring similarity by correlation with the automatic penalization of genes whose seemingly periodic variation is in the range of their measurement error. Note that in Equation (3), σ^2_{g,t_k} is used to describe the variance for the total mRNA levels. These quantities still need to be defined. In our application, given merely 2 replicate measurements per gene and time point, we face the challenge that the number of observations is not sufficient to estimate the variances σ^2_{g,t_k} meaningfully from the 2 replicates alone. Therefore, we use a maximum-a-posteriori approach to regularize the gene-wise empirical variance by an estimate of the overall, intensity-dependent variance of a microarray. For the estimation of σ^2_{g,t_k}, we let $\log \bar{g}(t_k)$ be the mean of the replicates $\log g(t_k, i)$, $i \in I$. We assume that the replicate measurements $\log g(t_k, i)$ are i.i.d. samples from a Gaussian distribution,

$$\log g(t_k, i) \sim \mathcal{N}(\log g(t_k, i);\, \log \bar{g}(t_k), \sigma^2_{g,t_k,})\ ,\ i \in I$$

For each time point, we calculate a global, intensity-dependent estimate of the variance by fitting a loess curve $m_{t_k}(.)$ [1] to the point set $(\bar{g}(t_k), var(g(t_k, i); i \in I))$, $g \in G$. Here, $var(g(t_k, i); i \in I)$ denotes the empirical variance.

We assume a Gamma prior on σ^2_{g,t_k} , given by

$$\sigma_{g,t_k} \sim \Gamma\left(\sigma_{g,t_k},\, k = k(\bar{g}(t_k)), \theta = \theta(\bar{g}(t_k))\right) \propto (\sigma_{g,t_k})^{k-1} \exp(-\frac{\sigma_{g,t_k}}{\theta}) \tag{4}$$

(where $\gamma(k)$ is the Gamma function). The shape parameter k and the scale parameter θ are chosen such that the expectation value of $\Gamma(\sigma;\, k, \theta)$ equals $m(\log \bar{g}(t_k))$, and its variance equals a parameter ν which is set to the mean of the squared residuals of the loess fit. This is achieved by letting

$$k = \frac{m(\log \bar{g}(t_k))^2}{\nu} \quad , \quad \theta = \frac{\nu}{m(\log \bar{g}(t_k))} \tag{5}$$

The regularized standard deviation is taken as the maximum a posteriori estimate

$$\sigma^{reg}_{g,t_k} = \arg\max_{\sigma_{g,t_k}} \left[\prod_{i \in I} \mathcal{N}(\log g(t_k, i);\, \log \bar{g}(t_k), \sigma^2_{g,t_k,}) \cdot \Gamma\left(\sigma_{g,t_k},\, k = k(\bar{g}(t_k)), \theta = \theta(\bar{g}(t_k))\right) \right]. \tag{6}$$

To safely guard against biases in the low intensity range, we additionally assume a minimum level for σ^{reg}_{g,t_k}, given by the 25% quantile of the respective residuals distribution.

1.3 Definition of periodic and non-periodic test functions

Definition of periodic test functions. Commonly, a gene g is called periodically expressed with period $\lambda' \in (0, \infty)$ and phase $\varphi \in [0, 2\pi]$ if its expression (in one cell) can be approximated, up to linear rescaling, by a cosine function

$$f(t) = cos(2\pi \cdot \frac{t}{\lambda'} - \varphi) \tag{7}$$

The phase φ describes the time at which g assumes its maximum expression divided by the cell cycle length; φ will therefore be also called the relative peak time. Accordingly, we call $\frac{\varphi}{2\pi} \cdot \lambda'$ the (absolute) peak time of g.

We wish to be less restrictive with respect to the shape of the periodic function. We use a slightly more general definition of a periodic gene. Let $\langle x \rangle$ the remainder x modulo 2π, i.e. the smallest non-negative number such that $x = \langle x \rangle + 2\pi z$ for some integer z. Let $\psi : [0, 2\pi] \to [0, 2\pi]$ be a monotonically increasing bijection of the unit interval. We consider a gene periodically expressed with period λ', phase φ and shape ψ if its expression can be approximated, up to linear rescaling, by a function $f = f(t;\, \lambda', \varphi, \psi)$,

$$f(t;\, \lambda', \varphi, \psi) = cos(\psi \left\langle 2\pi \cdot \frac{t}{\lambda'} \right\rangle - \varphi) \tag{8}$$

Note that fixing ψ to the identity function yields the original notion of a periodic gene (Equation 7).

We are measuring the population average of a large number of cells. Not all cells proliferate at exactly the same speed. We assume that the cell cycle period length in the sample is not constant for individual cells in the sample, it is distributed according to a random variable $\lambda' = \lambda'(\lambda, \sigma)$ with mean period length λ and a standard deviation of σ. The measured expression of a periodic gene within our sample population will therefore resemble, up to linear rescaling, a function

$$\gamma(t;\, \lambda, \varphi, \psi, \sigma) \quad = \quad \int f(t;\, \lambda', \varphi, \psi)\, d\lambda'(\lambda, \sigma) \tag{9}$$

We finally arrive at the definition of the family of periodic test functions, given by

$$\begin{aligned} \mathcal{F} \quad = \quad \{ \; & a \cdot \gamma(t;\, \varphi, \psi, \lambda, \sigma^2) + b \mid \\ & \varphi \in [0, 2\pi),\; \psi : [0, 2\pi] \to [0, 2\pi] \text{ a monotonically increasing bijection}, \\ & \lambda \in (0, \infty),\; \sigma^2 \in (0, \infty),\; a, b \in \mathbb{R} \} \end{aligned} \tag{10}$$

Choice of period length distribution. We tested three different classes of period length distributions for λ'. We scaled the parameters of the respective distributions such that they all have an

expectation value of λ and a variance of σ^2. First, we chose a Gaussian distribution that has been cropped to the interval $[20, 200]$,

$$\lambda' \sim U_{[20,200]} * \mathcal{N}(\text{mean} = \lambda,\ \text{variance} = \sigma^2)\ , \tag{11}$$

The cropping was necessary to avoid negative cell cycle times. Secondly, we chose a log-normal distribution

$$\lambda' \sim \mathcal{LN}(\text{logmean} = ln\lambda - \tau/2,\ \text{logsigma} = \sqrt{\tau})\ , \tag{12}$$

with $\tau = ln(\sigma^2/\lambda^2 + 1)$ and thirdly, we selected a Gamma distribution

$$\lambda' \sim \text{Gamma}(\text{shape} = \frac{\lambda^2}{\sigma^2},\ \text{scale} = \frac{\sigma^2}{\lambda}) \tag{13}$$

It turns out that the the mean and standard deviation of the cell cycle length distribution λ' are enough to determine the dampening of the test function $\gamma(t;\ \lambda, \varphi, \psi, \sigma)$ up to irrelevant fluctuations. No matter which of the above distribution classes we chose, the results were almost identical (Fig. S2), so we decided to use the log-normal distribution henceforth.

Definition of non-periodic test functions. Our goal is to discriminate periodic genes from non-periodic genes. To avoid false positive periodicity calls, the complementary set of non-periodic test functions should exhaustively cover time courses that a non-periodic gene can assume. Most often, a non-periodic gene has constant expression over time. Alternatively, due to continuous changes in the experimental conditions, non-periodic genes may show a constant drift, i.e., they are monotonically increasing or decreasing. There might also be genes that have one extraordinarily high / low peak at exactly one time point (in particular at $t = 0$). This might be due to a failure of the measurement, or due to synchronization at the beginning of the time course. We therefore define a family of non-periodic prototype test functions, consisting of the constant null function τ^0, a linearly increasing function τ^+, a linearly decreasing function τ^-, and the delta functions $\delta_k^+(t) = \begin{cases} 1 & \text{if } t = t_k \\ 0 & \text{else} \end{cases}$ and $\delta_k^-(t) = \begin{cases} -1 & \text{if } t = t_k \\ 0 & \text{else} \end{cases}$, $k = 1, ..., K$. We define the family $\overline{\mathcal{F}}$ of non-periodic functions as the set of all affine-linear transforms of the prototype test functions.

1.4 Kernel representation of the estimation problem.

Parameter estimation by MoPS can be cast as a regularized kernel regression problem. Given a gene expression measurement time course $g = (g(t_1), ..., g(t_K))$, our objective is the reconstruction of what we call a 'wobblet' $w = w(t)$, the expression time course of that gene in a single cell along one cell cycle as it would be obtained by error-free measurements. Here, $t \in [0, 1]$ denotes the time relative to the cell cycle length. Cell synchronization at the start of the experiment may be imperfect, and the time λ' for proceding through the cell cycle may differ slightly for each cell from the mean cell cycle time λ. Thus for each time t, we are observing a specific mixture of cells in different 'individual' relative cell cycle times s. Let $k = k(s, t)$ denote a so-called kernel function describing the individual cell cycle time distribution (the density function of s) at observation time t. Given the wobblet w and the kernel k, the predicted time course $\gamma = \gamma(t)$ of the cell mixture is given by

$$\gamma(t) = \int_0^1 w(s) \cdot k(s, t) \cdot ds \tag{14}$$

For an illustration, see Figure S3. Using γ, the measurements vector g is approximated by an affine transform of $(\gamma(t_1), ..., \gamma(t_K))$ via linear regression, i.e., $g \sim a \cdot (\gamma(t_1), ..., \gamma(t_K)) + b$ for suitable $a, b \in \mathbb{R}$.

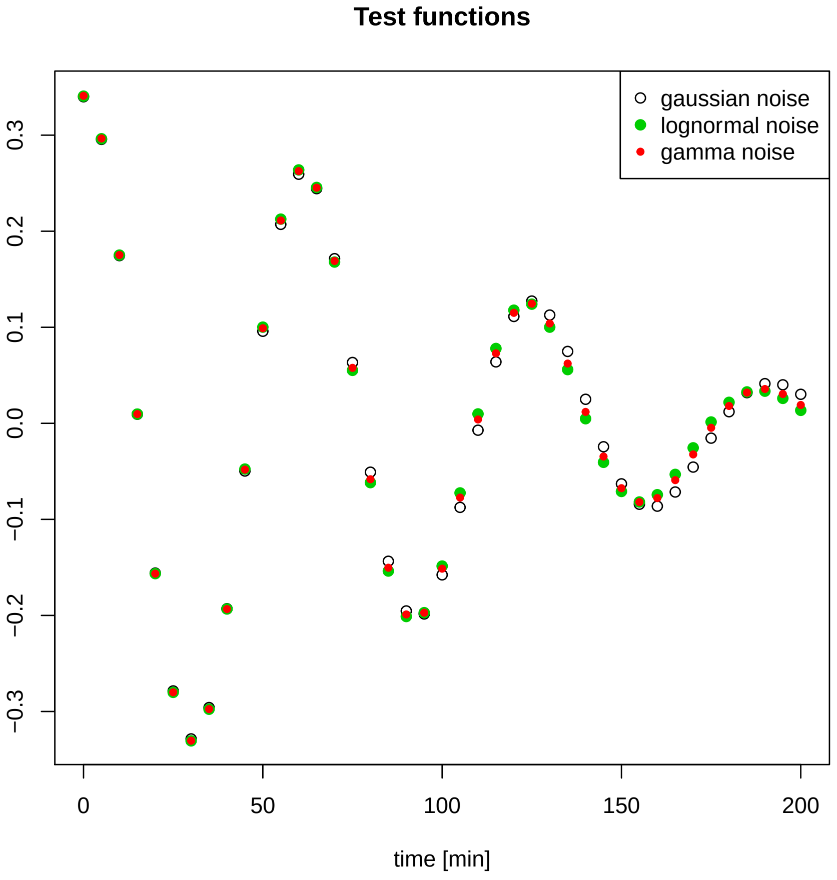

Figure 2: Dampening of a sine wave oscillation due to synchrony loss. Shown are the dampened curves $\gamma(t; \lambda, \varphi, \psi, \sigma)$ of a sine wave ($\varphi = 0$, $\psi = id$) resulting from three different cell cycle length distributions $\lambda' = \lambda'(\lambda, \sigma)$, all of which have the same mean ($\lambda = 62.5$min) and standard deviation ($\sigma = 7$min). The values for mean and standard deviation match the situation in our application. The shapes of the cell cycle length distributions λ' were chosen as a $[0, \infty)$ truncated Gaussian (circles), a log-normal distribution (green dots), and a Gamma distribution (red dots).

This approach is common for parametric periodicity screens [6, 5]. Various methods differ in the way the functions $w(s)$ and are parametrized and inferred. [6] propose a Fourier base of degree 3 for modeling of the wobblets, and [5] compute wobblets as an L1(Lasso)-regularized approximation using a Symlet wavelet base of degree 5. We model wobblets by time-shifted cosine functions for which the x-axis is stretched or compressed differently at different time points (see Figure 1 in the main text and Supplements 1.5 for a precise definition). Hartemink et al. explicitly construct the kernel function $k(s,t)$ from FACS measurements of fluorescently labeled cells at a given time t which allow the estimation of the density function $k(s,t)$. This is of particular advantage if the synchronization at the start of the time course is weak. We do not have access to similar data for our experiments, however we use a stringent synchronization and assume that cells are perfectly synchronized at t=0. [6] assume that the inverse of the individual, single cell period lengths follows a normal distribution, which ultimately determines $k(s,t)$. Their parametrization of w and k allows a convenient reduction of the inference problem to a simple linear regression. This is not possible for our wobblets, but it gives us the freedom to derive our kernel $k(s,t)$ from the slightly more realistic assumption that single cell period lengths follow a log-normal distribution (see Supplements 10). Because a wobblet is estimated from all available measurements of a 200 min time course, its peak time can potentially be resolved at a higher resolution than our sampling rate (5 min), and it is likely more precise than the time of maximum raw or kernel-smoothed expression. Wobblets therefore capture periodic expression characteristics robustly and can be used to group periodic genes.

The representation of γ in Equation (9) can be transformed into a kernel representation. This has practical consequences for the parameter learning strategies. Denote by $\rho(r;\lambda,\sigma^2)$ the density function of λ'.

$$\begin{aligned}
\gamma(t;\,\lambda,\varphi,\psi,\sigma) &= \int_{\mathbb{R}} f(t;\,\lambda',\varphi,\psi)\,d\lambda'(\lambda,\sigma^2) \\
&= \int_{\mathbb{R}} cos(\psi\left\langle 2\pi\cdot\frac{t}{\lambda'}\right\rangle-\varphi)\,d\lambda'(\lambda,\sigma^2) \\
&= \int_{\mathbb{R}} cos(\psi\left\langle 2\pi\cdot\frac{t}{r}\right\rangle-\varphi)\cdot\rho(r;\lambda,\sigma^2)\,dr \\
&\overset{s=\frac{t}{r}}{=} \int_{\mathbb{R}} cos(\psi\left\langle 2\pi\cdot s\right\rangle-\varphi)\cdot\rho(\frac{t}{s};\lambda,\sigma^2)\cdot ts^{-2}\,ds \\
&= \int_0^1 w(s;\varphi,\psi)\cdot k(s,t;\lambda,\sigma)\,ds
\end{aligned} \tag{15}$$

for $w(s;\varphi,\psi)=cos(\psi\left\langle 2\pi\cdot s\right\rangle-\varphi)$ and the kernel $k(s,t;\lambda,\sigma)=\sum_{j=0}^{\infty}\rho(\frac{t}{s+j};\lambda,\sigma^2)\cdot ts^{-2}$. Thus, γ has a kernel representation as defined in Equation (14). For practical reasons, it is convenient that we also achieve a separation of the parameter inference problem. The wobblet w only depends on the gene-specific parameters peak time φ and the shape ψ, whereas the kernel k only depends on the global parameters mean cell cycle length λ and synchrony loss σ^2.

1.5 Parametrization of and screening for periodic genes

Maximum likelihood estimation in $\mathcal{F}$. The infinite family of periodic test functions $\mathcal{F}$ is parameterized by the tuple $(a,b,\lambda,\varphi,\psi,\sigma^2)$ (Equation (10)). Given a time series g, our task is to find the maximum likelihood estimate $f_g = argmin_{\gamma\in\mathcal{F}} l(g,\gamma)$. We need to explain how we perform maximum likelihood search in $\mathcal{F}$. To that end, we construct a finite set of "prototype" functions $\mathcal{G}$, whose affine hull $\bar{\mathcal{G}}=\{a\gamma+b \mid \gamma\in\mathcal{G},\ a,b\in\mathbb{R}\}$ is assumed to lie sufficiently dense in $\mathcal{F}$. An approximation of the

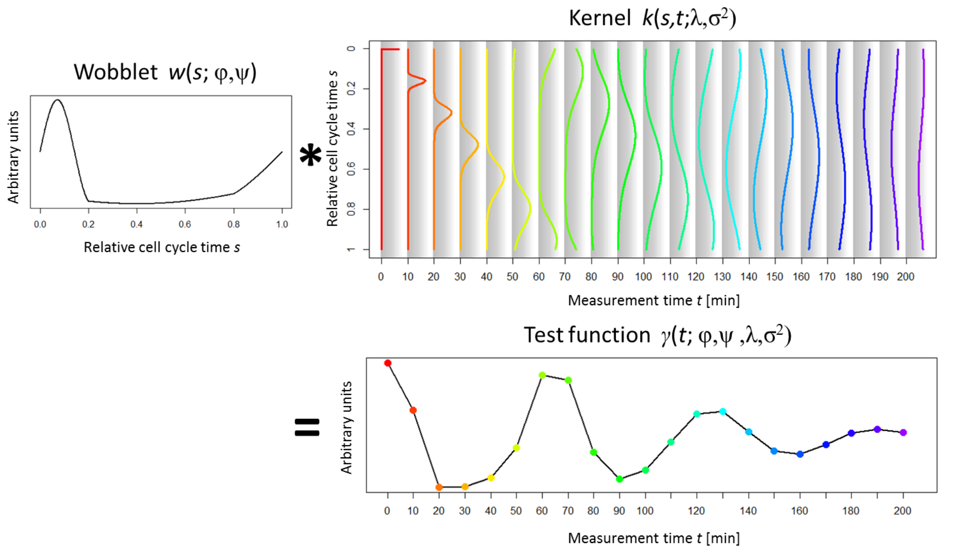

Figure 3: Wobblets kernel representation. The test function $\gamma = \gamma(t, \varphi, \psi, \lambda, \sigma)$ is given as the convolution $\gamma = w * k$ of a wobblet $w = w(s; \varphi, \psi)$ with with a kernel function $k = k(s, t; \lambda, \sigma)$. In our example here, we chose $\varphi = 0$, $\psi = id$, $\lambda = 62.5min$ and $\sigma =$ 7min. For each time point t, the function $d_t(s) = k(s, t; \lambda, \sigma)$ is the cell cycle phase distribution of the individual cells in a population at time t. The functions $d_t(s)$ for $t =$ 5, 10, 15, ..., 200min are shown as a colored lines in the box visualizing the kernel function k. At $t = 0$, $d_t(s)$ is sharply peaked, the cell population is perfectly synchronized. With increasing t, $d_t(s)$ approaches the constant function, i.e., our population contains an even mixture of cell cycle times.

maximum likelihood estimate in $\mathcal{F}$ is then given by

$$\begin{aligned} f_g &= argmin_{\gamma\in\mathcal{F}} l(\gamma;\, g) \;\approx\; argmin_{\gamma\in\bar{\mathcal{G}}} l(\gamma;\, g) \\ &= argmin_{\gamma\in\mathcal{G}} \left[argmin_{(a,b)} l(a\gamma+b;\, g)\right] l_2 = \arg\max \end{aligned} \tag{16}$$

The minimization problem for (a, b), given γ, can be solved analytically by a weighted linear regression, using the error model in (Equation (3)):

$$g(t_k) \sim \gamma(t_k) \quad , \text{ with weights } \sigma_{g,k}^{-2}\ ,\ k = 1, ..., K \tag{17}$$

The slope of the regression line determines a, and the intercept determines b. The minimization over $\gamma \in \mathcal{G}$ is done by exhaustive search. The set $\mathcal{G}$ is defined as $\mathcal{G} = \{\psi(t;\, \lambda, \varphi, \psi, \sigma^2) \mid \lambda, \varphi, \psi, \sigma^2$ taken independently from a representative grid$\}$. The grid $\mathcal{G}_\lambda$ for λ runs from 30min to 90min in steps of 2.5min. The grid $\mathcal{G}_{\sigma^2}$ for the variance σ^2 runs from 1min^2 to 15min^2 by steps of 1min^2, and the grid $\mathcal{G}_\varphi$ for the peak time φ runs from 0 to $2\pi \cdot \frac{39}{4}$ in 40 equidistant steps. ψ runs through a representative set of piecewise linear, monotonically increasing functions that are parametrized by a vector $y = (y_1, ..., y_{r-1})$ in the following way: Let $r = 4$, and let $t_j = j/r$, $j = 0, ..., r$. Define $\psi(t;\, y)$ as the piecewise linear function which linearly interpolates the points (t_j, y_j), $j = 0, ..., r$ (set $(t_0, y_0) = (0, 0)$ and $(t_r, y_r) = (1, 1)$). Formally,

$$\psi(t;\, y) = r \cdot [(t - t_{j-1}) \cdot y_{j-1} + (t_j - t) \cdot y_j] \quad \text{if } t \in [t_{j-1}, t_j]$$

The values $y_1, ..., y_{r-1}$ are chosen from a finite grid

$$\mathcal{G}_\psi = \{(y_1, ..., y_{r-1}) \mid y_j \in \{\frac{0}{d}, \frac{1}{d}, ..., \frac{d}{d}\};\, y_1 \le y_2 \le ... \le y_{r-1}\}\ ,$$

for a given grid density d (we chose $d = 5$). In this way, a function $f \in \mathcal{F}$ is completely characterized by the tuple

$$(\varphi, y = (y_1, ..., y_{r-1}), \lambda, \sigma^2, a, b) \in \mathcal{G} = \mathcal{G}_\varphi \times \mathcal{G}_\psi \times \mathcal{G}_\lambda \times \mathcal{G}_{\sigma^2} \times \mathbb{R} \times \mathbb{R}$$

With our choice of $r = 4$, these are in total 7 parameters.

Note however, that the parameters are redundant: Assume that r is an even number. Let $\psi = \psi(.;\, (0, y, 1))$ be parametrized by a vector $y \in \mathcal{G}_\psi$ as described above. Define $y' = (y'_1, ..., y'_{r-1})$ by $y'_j = \begin{cases} y_{j+r/2} - y_{r/2} & \text{if } j = 1, ..., \frac{r}{2} - 1 \\ y_{j-r/s} - y_{r/2} + 1 & \text{if } j = \frac{r}{2}, ..., r-1 \end{cases}$ (check that $y' \in \mathcal{G}_\psi$). By elementary calculations, it can be shown that

$$f(t;\, \lambda, \varphi, \psi(.;\, (0, y, 1))) = -f(t + \frac{\lambda}{2};\, \lambda, \varphi + \pi, \psi(.;\, (0, y', 1))$$

I.e., a phase shift by $\frac{\lambda}{2}$ can be described by a re-parametrization of the shape parameters, and by switching the sign. In other words, the parameter tuples $(\varphi, y, \lambda, \sigma^2, a, b)$ and $(\varphi + \pi, y', \lambda, \sigma^2, -a, b)$ describe identical test functions. Therefore, we only need to screen for φ values between 0 and π. In order to assign the correct peak time afterward, we simply need to check the sign of a in the linear regression. If $a \ge 0$, we keep the parameter set $(\varphi, y, \lambda, \sigma^2, a, b)$. If a is negative, the corresponding set $(\varphi + \pi, y', \lambda, \sigma^2, -a, b)$ is the one with the correct peak time.

Maximum likelihood estimation in $\bar{\mathcal{F}}$. Since $\bar{\mathcal{F}}$ is the affine hull of a finite set of prototype functions, we proceed as in (Equation (16)). The maximum likelihood estimate in $\bar{\mathcal{F}}$ can be calculated exactly as

$$\begin{aligned} \bar{f}_g &= argmin_{\gamma\in\bar{\mathcal{F}}} l(\gamma;\, g) T \\ &= argmin_{\gamma\in\text{prototypes}} \left[argmin_{(a,b)} l(a\gamma+b;\, g)\right] \end{aligned} \tag{18}$$

Initial screen for periodic genes. The set $\tilde{P}$ of periodic genes is defined as the set of genes g for which the (log) likelihood ratio statistic $log\frac{L(f_g; g)}{L(\overline{f}_g; g)}$ exceeds some threshold value t_{min}. Genes that are not in $\tilde{P}$ are considered non-periodic. Assuming a fraction of 1/10 of periodic genes among all genes, t_{min} is determined by requiring that our criterion for periodicity have a false discovery rate of $\alpha = 0.05$ (see 2.3). In this way, we identify genes that are periodically expressed with high confidence and can estimate the mean cell-cycle length and variation for each time series.

Refined screening to determine gene-specific parameters.

We estimate two gene-independent parameters, the mean cell cycle length λ and the variance σ^2 of the cell cycle length distribution in the initial screen from high-confidence periodic genes. For fixed λ, σ^2, let $h_{g,\lambda,\sigma^2} = \hat{a}_g \cdot \gamma(t;\, \lambda, \hat{\varphi}_g, \hat{\psi}_g, \sigma^2) + \hat{b}_g$ be the maximum likelihood approximation of g under the constraint that $\lambda_g = \lambda$ and $\sigma_g^2 = \sigma^2$, i.e.,

$$(\hat{a}_g, \hat{b}_g, \hat{\varphi}_g, \hat{\psi}_g) = argmin_{(a_g,b_g,\varphi_g,\psi_g)} l(a_g \cdot \gamma(t;\, \lambda, \varphi_g, \psi_g, \sigma^2) + b_g;\, g) \tag{19}$$

To determine the most likely global parameters $\hat{\lambda}$, $\hat{\sigma}^2$, we solve

$$(\hat{\lambda}, \hat{\sigma}^2) = argmin_{(\lambda,\sigma^2)} \sum_{g \in \tilde{P}} l(h_{g,\lambda,\sigma^2};\, g) \tag{20}$$

Note that the sum in Equation (20) is taken only over the initially defined periodic genes, because these are the candidates that are informative for the estimation of the global cell cycle parameters.

A gene g is called periodic, if

$$log\frac{L(h_{g,\hat{\lambda},\hat{\sigma}^2};\, g)}{L(\overline{f}_g;\, g)} > t_{min} \tag{21}$$

The set of all periodic genes is denoted by P. Genes that are defined as significant periodic in the refined screen are implicitly also significant periodic in the initial screen ($\tilde{P} \subseteq P$), since $L(h_g;\, g) \geq L(h_{g,\hat{\lambda},\hat{\sigma}^2};\, g)$.

Accounting for replicate experiments. Our cell cycle experiment was done in two biological replicate time series, we do not only have one, but two time series, $(g^{(r)}(t_k))_{k=1,\dots,K}$, $r \in R = \{1, 2\}$, for each gene g . We noticed that there are slight differences in the cell cycle length, thus we estimate two global parameter sets $\lambda^{(r)}$, $(\sigma^{(r)})^2$, $r = 1, 2$. The gene-specific parameters $a_g^{(r)}$, $b_g^{(r)}$ are estimated separately for each experiment, because it is not unlikely that there are slight differences in the magnitude of regulation due to slightly changed environmental conditions. The parameters $\hat{\varphi}_g$, $\hat{\psi}_g$ that determine the shape of the test function however are assumed to be common to all replicates. Finally, our screening procedure for periodic genes can be stated:

Definition of the Periodicity score and screen for periodic genes.

- Input: Expression time series measurements $g^{(r)} = (g^{(r)}(t_k))_{k=1,\dots,K}$, $g \in G$, $r \in R$.
- For $g \in G$, $r \in R$, estimate the maximum likelihood fit of $g^{(r)}$ in $\mathcal{F}$ resp. $\overline{\mathcal{F}}$,

$$h_g^{(r)} = argmin_{\gamma \in \overline{\mathcal{G}}} l(\gamma;\, g^{(r)}) \in \mathcal{F} \quad , \quad \overline{f}_g^{(r)} = argmin_{\gamma \in \overline{\mathcal{F}}} l(\gamma,\, g^{(r)}) \in \overline{\mathcal{F}}$$

- Initital screen: determine a threshold t_{min}, and find all periodic genes $\tilde{P}^{(r)}$ in replicate $r \in R$,

$$\tilde{P}^{(r)} = \{g \in G \mid log\frac{L(h_g^{(r)};\, g^{(r)})}{L(\overline{f}_g^{(r)};\, g^{(r)})} > t_{min}\}$$

- For each replicate $r \in R$, calculate the global parameters $\hat{\lambda}^{(r)}$, $(\hat{\sigma}^{(r)})^2$ by

$$(\hat{\lambda}^{(r)}, (\hat{\sigma}^{(r)})^2) = argmin_{(\lambda toidentify, \sigma^2)} \sum_{g \in \tilde{P}^{(r)}} l(h^{(r)}_{g,\lambda,\sigma^2}, g^{(r)}) \ , \tag{22}$$

  where $h^{(r)}_{g,\lambda,\sigma^2}$ is the maximum likelihood approximation of $g^{(r)}$ in $\overline{\mathcal{G}}$ under the constraints $\lambda^{(r)}_g = \lambda$, $(\sigma^{(r)})^2 = (\sigma^{(r)})^2$ (see Equation (19)).

- Refined screening: for each gene $g \in G$, calculate $\hat{\varphi}_g$, $\hat{\psi}_g$, $a^{(r)}_g$, $b^{(r)}_g$, $r \in R$ by

$$(\hat{\varphi}_g, \hat{\psi}_g, a^{(r)}_g, b^{(r)}_g;\ r \in R) = argmin_{(\varphi_g, \psi_g, a^{(r)}_g, b^{(r)}_g;\, r \in R)} \sum_{r \in R} l(a^{(r)}_g \cdot \gamma(t;\ \hat{\lambda}^{(r)}, \varphi_g, \psi_g, \hat{\sigma}^2) + b^{(r)}_g;\ g^{(r)}) \tag{23}$$

  Let $f^{(r)}_g = \hat{a}^{(r)}_g \cdot \gamma(t;\ \hat{\lambda}^{(r)}, \hat{\varphi}_g, \hat{\psi}_g, (\hat{\sigma}^{(r)})^2) + \hat{b}^{(r)}_g$.

- Define the periodicity score $T(g)$ as

$$T(g) = \sum_{r \in R} log \frac{L(f^{(r)}_g;\ g^{(r)})}{L(\bar{f}^{(r)}_g;\ g^{(r)})} \tag{24}$$

- Define the set P of periodic genes, $P = \{g \in G \mid T(g) > |R| \cdot t_{min}\}$.

- Output: The set P of periodic genes, and a set of parameters $\{\hat{\lambda}^{(r)}, (\hat{\sigma}^{(r)})^2, \hat{\varphi}_g, \hat{\psi}_g, a^{(r)}_g, b^{(r)}_g;\ g \in G,\ r \in R\}$.

2 Application of MoPS to cell cycle cDTA data

2.1 Data exploration and quality control

Before performing high-level analyses, we did elementary quality checks. First, we calculated the relative deviation from the mean for all genes and all replicate samples (Figure 4 left plot). 95% percent of the replicate measurements have a relative deviation smaller than 0.4. The right plot in Figure 4 shows a scatterplot of a representative sample (labeled RNA, 50 min after synchronization).

Second, we visualized the pairwise correlations between the total respectively labeled measurements between all time points and between replicates. It turns out that the agreement between replicate measurements of corresponding time points is excellent. The mean correlation is 0.97 (min=0.93, max=0.99). The 3 periods of the cell cycle show as 3 diagonal regions of high correlation (Figure 5).

Third, we did a principal component analysis of the (total RNA) microarray measurements. Each time point is represented by its projection onto the two largets principal components. The periodicity of approximately 60min is evident. Further, the dampening of the oscillations make the later time points move closer to the center, which creates a spiral (Figure 6).

2.2 High precision estimates of mRNA synthesis rates and half lifes

We use all labeled and total measurements to calculate a high precision estimate of (steady-state) mRNA synthesis rates and half lifes. To achieve this, we simply ignore the time at which the sample was taken, and treat all samples as replicates. By doing so, we obtain 82 replicate measurements of

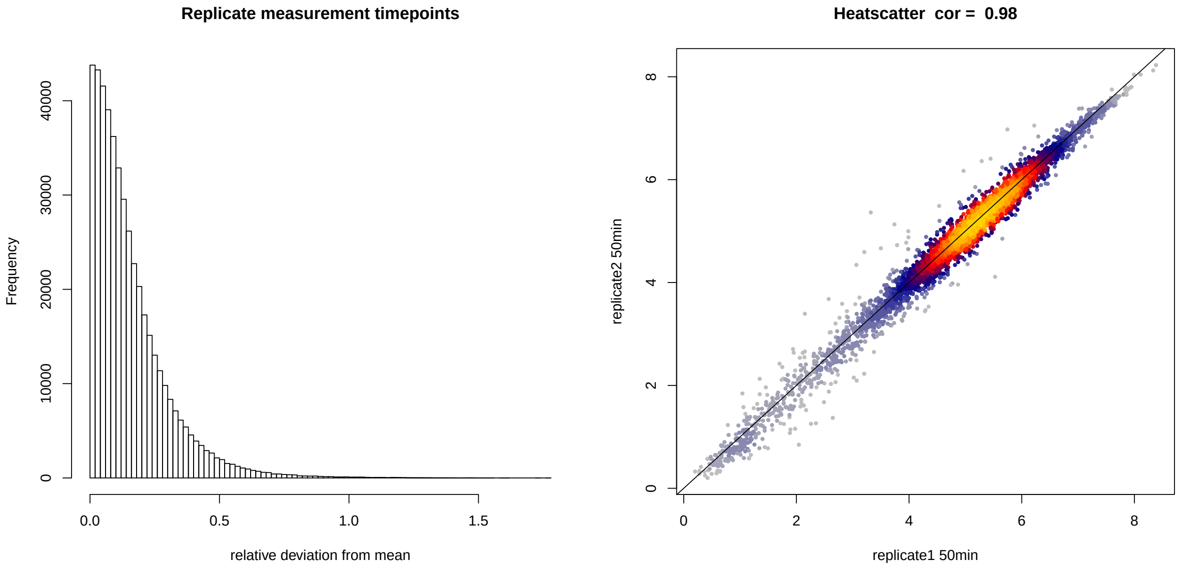

Figure 4: Comparison of replicate measurements. The histogram on the left shows the relative deviation from the mean for all measurements (labeled and total, all genes). As a representative example, the scatterplot on the right shows a comparison of the log labeled expression levels for all genes 50 minutes after synchronization in the two independent time series.

total and labeled mRNA expression respectively. We use this large number of samples to calculate high accuracy steady-state synthesis rates and half lifes with the statistical framework described in[12]. These estimates are provided as supplementary tables (Supplementary Table 4 and Supplementary Table 5). A comparison of our new estimates to the estimates from[12] shows an excellent agreement (Figure 7).

2.3 Significance of MoPS periodicity scores

MoPS computes a periodicity score for each gene and thus allows ranking of all genes according to their likelihood ratio to be periodically expressed respectively constantly expressed. However, there is no obvious way to assign significance to this score. We want to make use of existing knowledge derived from published studies about periodically expressed genes. To do this, we define a positive set and a negative set. The positive set comprises the top 200 periodic genes from Cyclebase [3] and the negative set consists of genes that have never been classified as cell-cycle regulated in any cell-cycle expression study considered [11, 4, 10]. The empirical distribution f of all MoPS scores is fitted by a mixture of the empirical distributions $f_+$and f_-scores of the positive respectively the negative set,

$$f \approx \mu \cdot f_+ + (1-\mu) \cdot f_- \quad ,$$

where the mixture coefficient $\mu \in [0,1]$ estimates the fraction of periodic genes among all genes (see main Figure 8). Fitting of μ was done be minimization of the Kolmogoroff-Smirnov statistic. μ, f_+ and f_- were then used to calculate the false discovery rate $FDR(c)$ as a function of the cutoff value c by

$$FDR(c) = \frac{(1-\mu) \cdot \int_c^\infty f_-(t)dt}{\int_c^\infty f(t)dt}$$

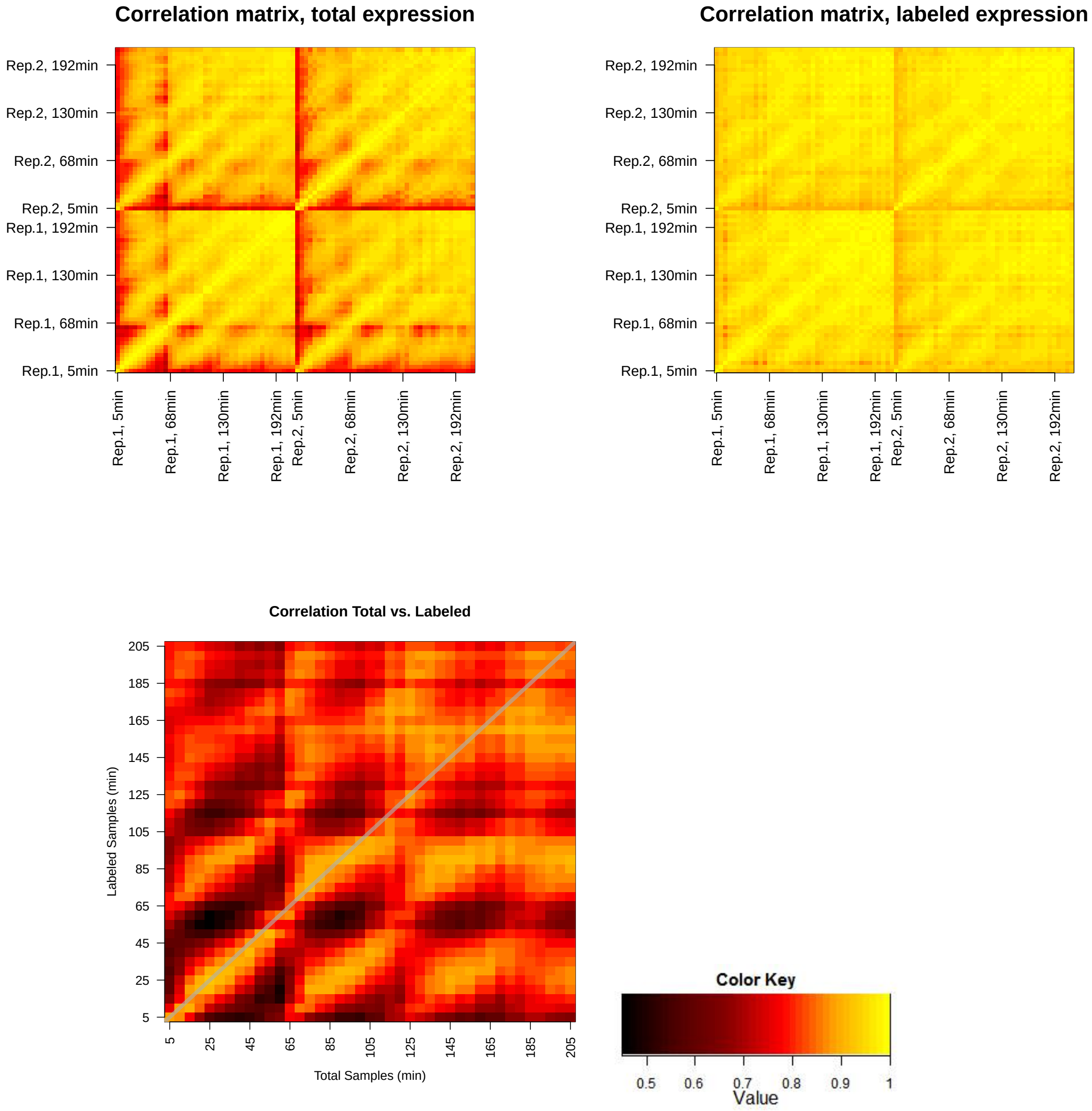

Figure 5: Correlation between time points and between replicates of the total RNA measurements (top left), the labeled RNA measurements (top right), and between total and labeled measurements of the first replicate (bottom). Correlation values are color-coded (bottom).

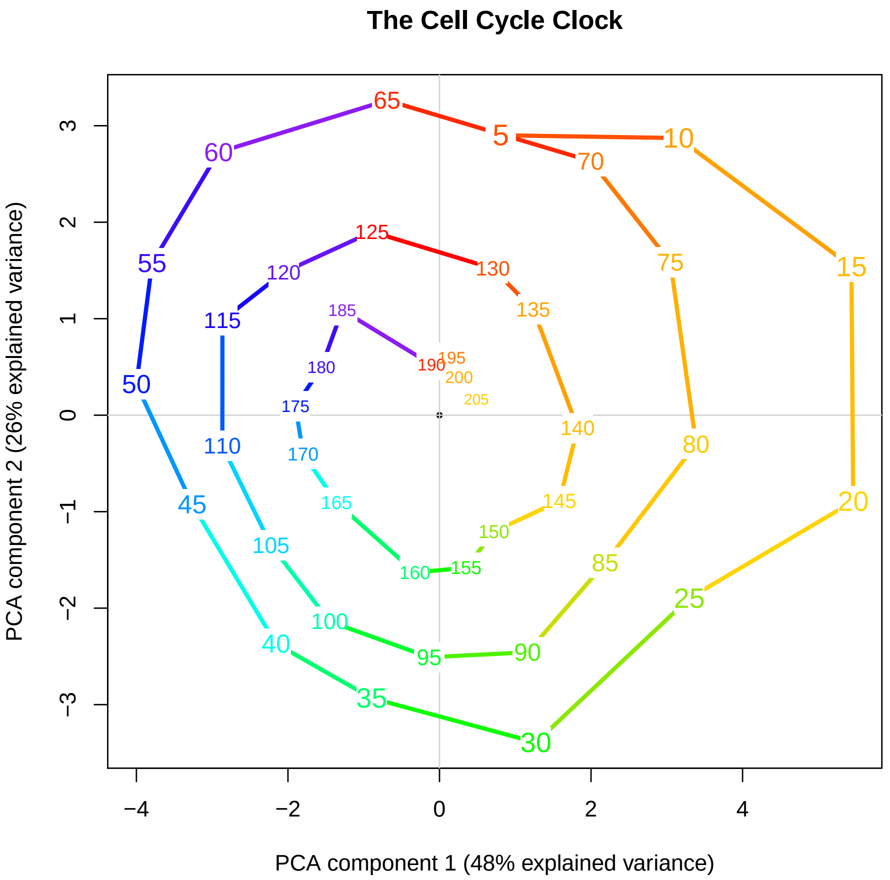

Figure 6: The yeast 'cell cycle clock'. Each point corresponds to the microarray measurements of one time point. Its coordinates are the projection of the corresponding expression vectors onto the two first principal components in a principal component analysis. Color coding is according to time in the cell cycle.

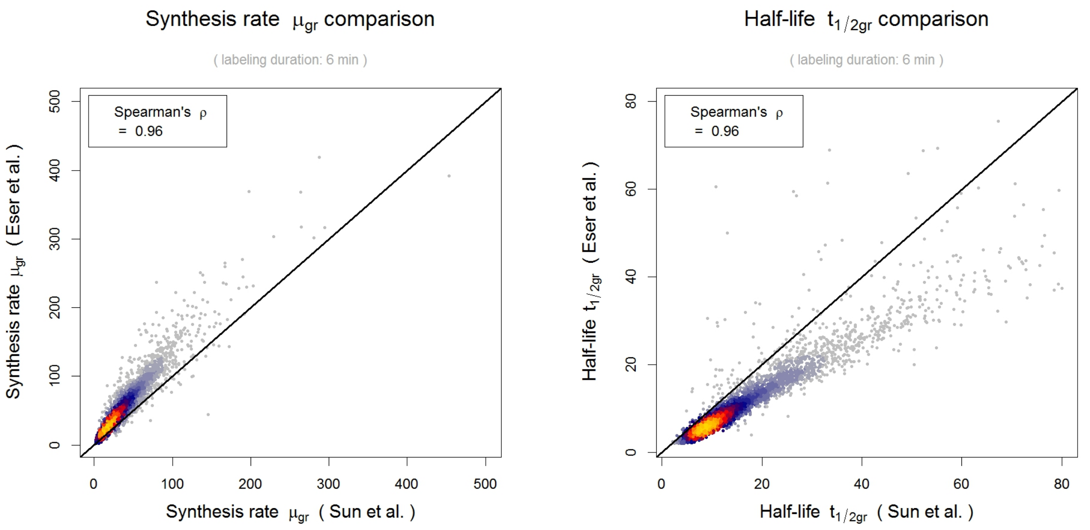

Figure 7: Comparison of cDTA derived genome-wide synthesis rates and half-lifes.

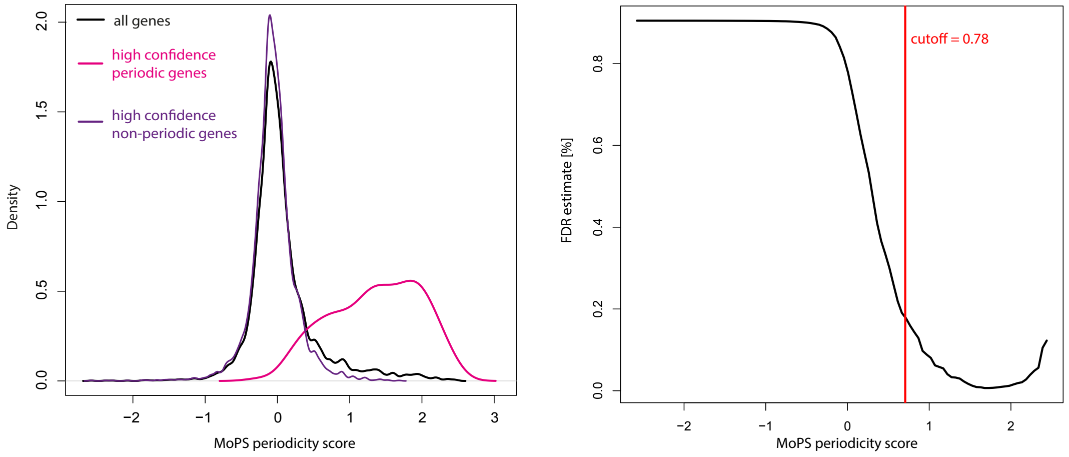

Figure 8: Periodicity score cutoff determination. (Left) The densities of MoPS derived periodicity scores for high confidence periodic, non-periodic and all genes are shown. (Right) A cutoff is estimated for a given false-discovery rate of 20%.

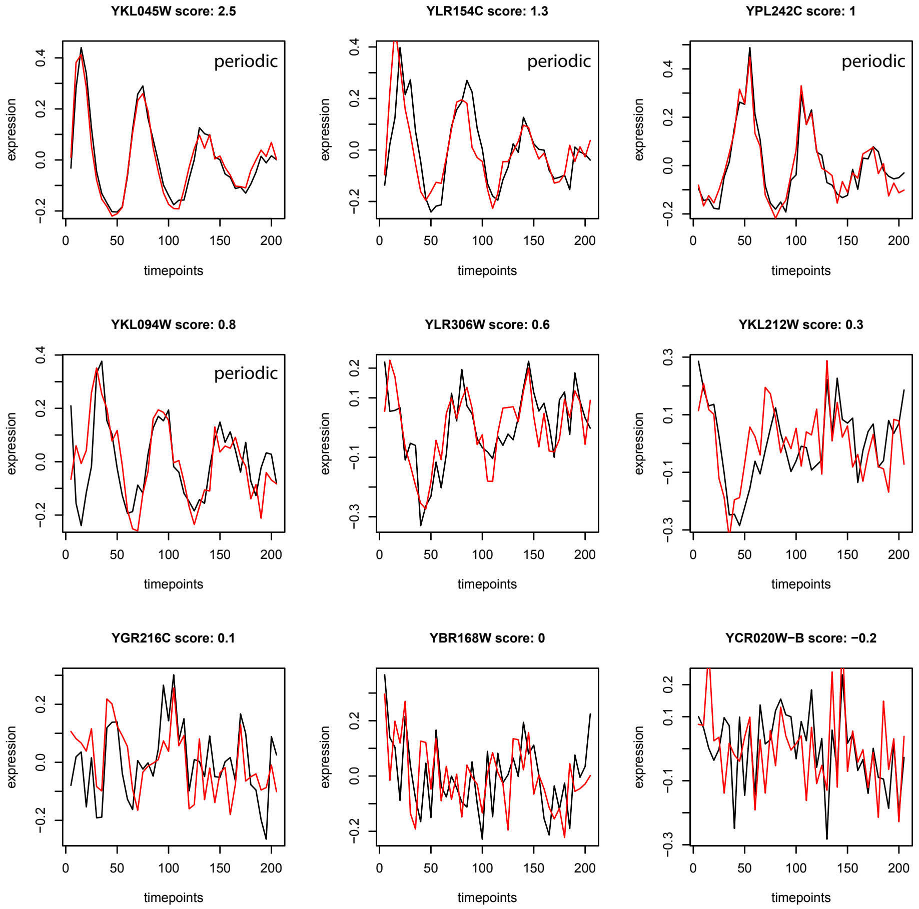

Figure 9: Examples of nine genes with various periodicity scores estimated with MoPS. Shown are total (black) and labeled (red) expression time courses (replicates averaged). All genes with a score above 0.78 are deemed periodic and considered for downstream analyses.

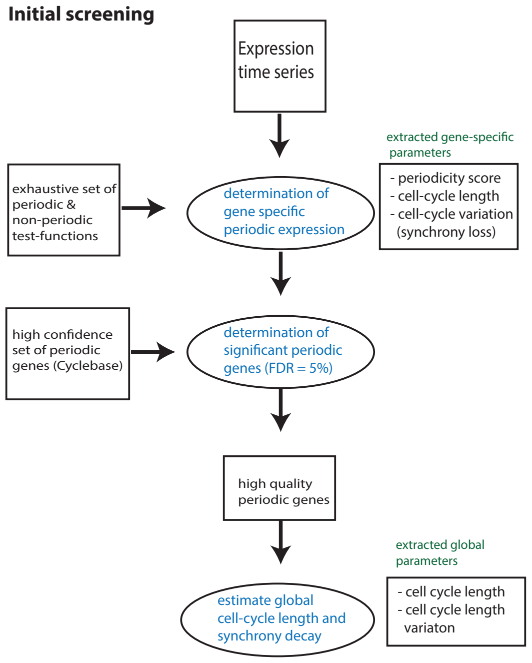

Figure 10: Initial screening for periodic genes. In an inital screen a periodicity score and characteristic parameters for each gene are estimated and used to determine the mean cell-cycle length and its variation in the population of synchronized cells.

2.4 Estimation of the global parameters cell-cycle length and variation

In an initial screen we fit periodic test-functions that represent different combinations of cell-cycle length (λ), cell-cycle length variation in the population (σ) and phase (φ) to each expression profile. Using a strict periodicity score cutoff (FDR $<$ 5%, scores with best fitting λ, σ for each gene) this results in a set of periodic genes for each dataset with associated loss for all examined λ, σ combinations. The globally best fitting λ and σ are then estimated by minimizing the overall loss for each combination over all genes (see Figure 10 and Section 1.5). The distribution of estimated gene-specific λ and σ agree well within each dataset and between datasets. λ values range from 55 to 65 minutes and σ are mostly estimated to be in the range of 4 to 8 minutes.

2.5 Estimation of gene-specific parameters

A second screening is then performed using the dataset-specific global parameters λ and σ together with a refined set of periodic test-functions which are constructed from a exhaustive combination of the gene-specific parameters (see Section 1.5 and Figure 1 B, main text). Expression time courses of all genes are fitted to those periodic test-functions, separately for each dataset. This refines the initial screening by estimating gene-specific characteristic parameters. (Figure 12, Section 1.5). The derived characteristic expression time courses, the timing of peak expression and periodicity score are highly correlated between replicates (see Figure 14).

Total and labeled mRNA time courses show a high correlation in periodicity scores (see 15). It is evident that genes with periodic labeled mRNA levels also exhibit periodic total mRNA levels. The calculation of a periodicity score cutoff separately for each dataset, results in 58 genes that are

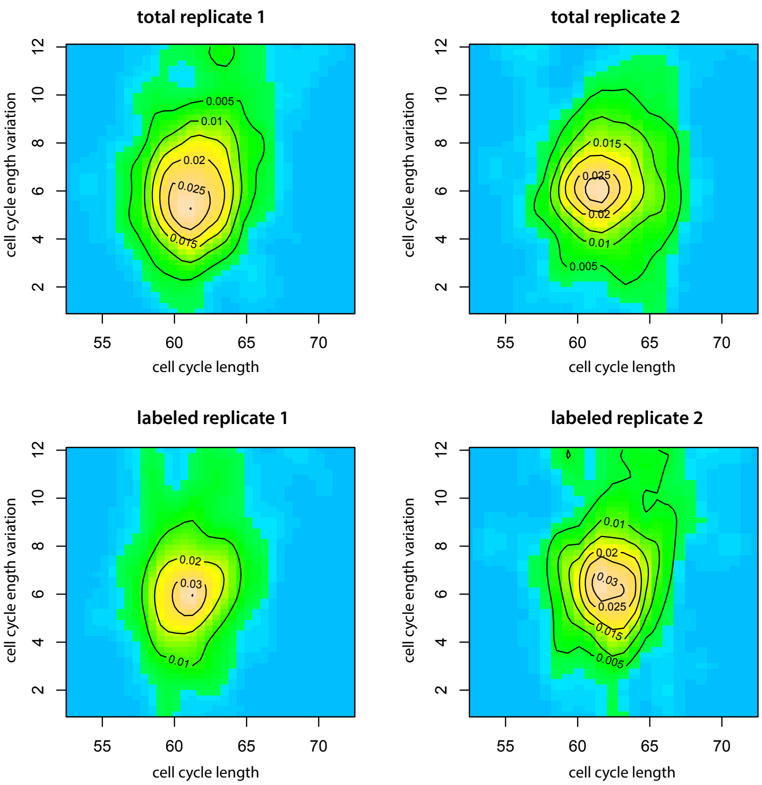

Figure 11: Cell-cycle length and variation distributions estimated in initial screen. For each time series, MoPS is used to estimate the best-fitting cell-cycle length and variation for each gene, which are shown as 2D density maps. The distributions are similar for all time series and show an increased density at a cell-cycle length of 60-63 min and variation of 5-8 min.

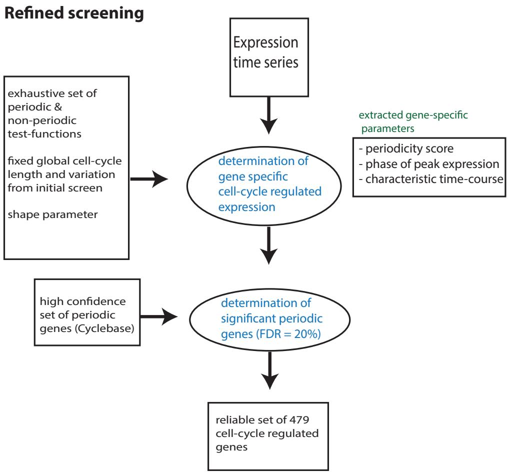

Figure 12: MoPS refined screening.

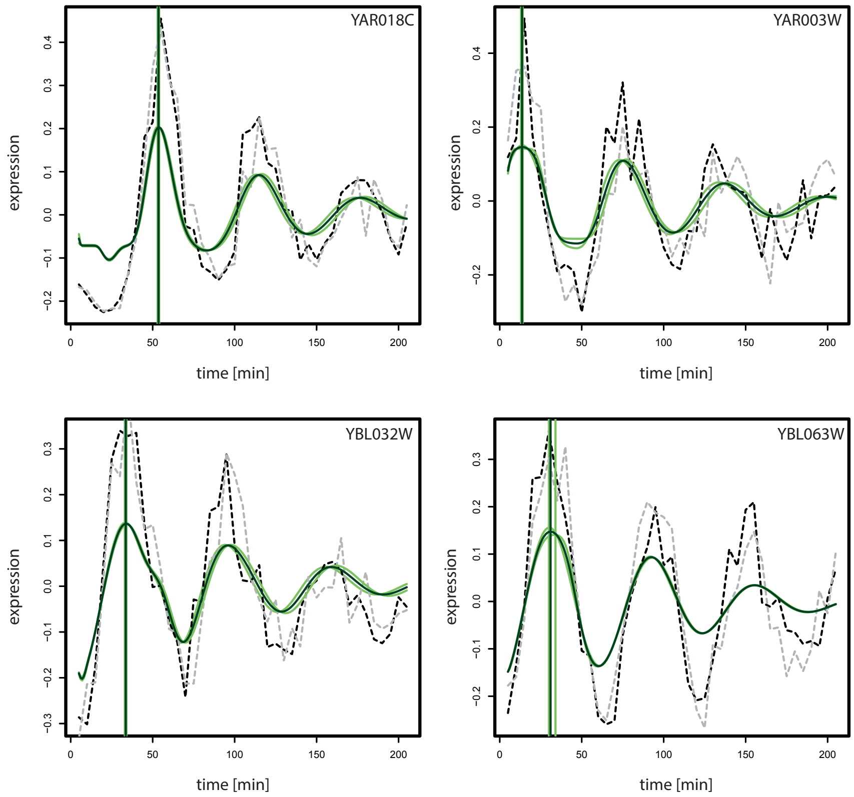

Figure 13: Examples of MoPS fits to replicate experimental time courses. Shown are replicate expression measurements (black and grey dotted lines) of selected genes together with the replicate-specific fitted characteristic time courses and timing of peak expression (light green). The estimates are averaged for further analyses (dark green). Note that the fitted curves are scaled to L2-norm 1.

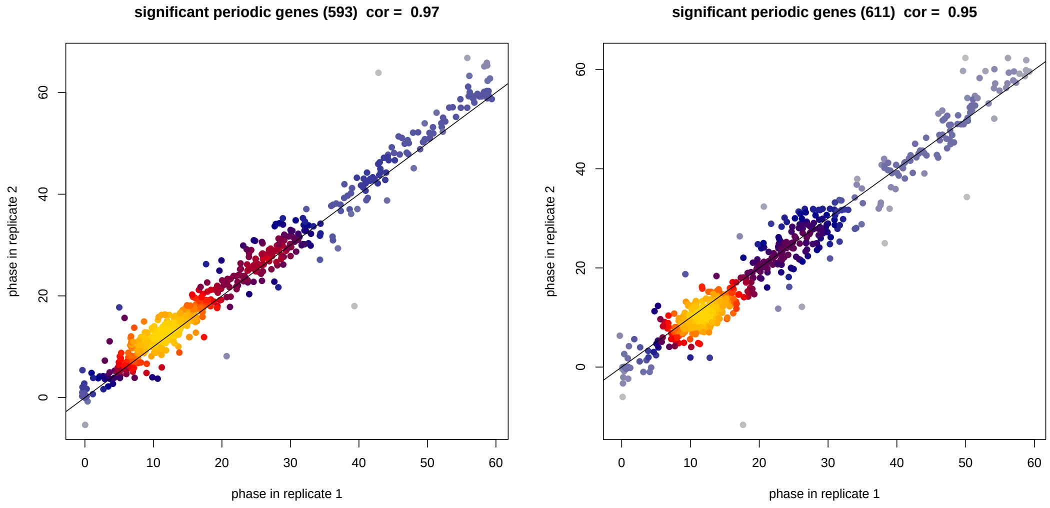

Figure 14: Correlation of timing of peak expression between replicates. Estimated phases of genes that are significant periodic are compared in Total (left) and Labeled (right). The pearson correlation coefficient of 0.97 and 0.95 for labeled and total respectively shows the excellent agreement.

significantly periodic in only one dataset but not in the other (25 genes only periodic in labeled, 33 genes only periodic in total). Visual inspection of their profiles, shows that the disagreement stems mainly from poor correlation among replicates or atypical expression profiles due to synchronization.

Since the periodicity scores of labeled and total datasets are highly similar for genes with positive scores (see Figure 15), we averaged the scores and estimated one cutoff to obtain one set of cell-cycle regulated genes. Controling the false dicovery rate at 20%, we derive a cutoff of 0.78, which results in 479 significantly cell-cycle regulated genes. For each gene, the best fitting 1 min resolution characteristic time course and its peak timing are averaged in total and labeled replicate time series. Examples of MoPS derived characteristic profiles are shown in Figure 16 and Figure 17.

2.6 Quantification of absolute mRNA abundance

The mean expression and amplitude is estimated for all 479 periodic genes by fitting their MoPS estimated characteristic time course to absolute mRNA concentrations. The minimzation problem is solved with linear regression (see section 1.5). This extends the MoPS estimated time courses by adding information about the absolute mRNA levels (Figure 18). Mean expression levels of non-periodic genes are determined by using the mean expression in the time course of the first cell cycle. We observe a very high correlation of absolute mean mRNA levels in replicates of total and labeled datasets (Figure 19).

2.7 Cyclins and histones peak timing define cell-cycle stages

The estimated median cell-cycle length of all four datasets of 62.5 min can now be used to find the boundaries of each cell cycle stage (G1,S,G2,M). We use the total mRNA peak timing of cyclins and histones which have a well defined cell-cycle specific timing of activity (see Table 1 and Figure 22).

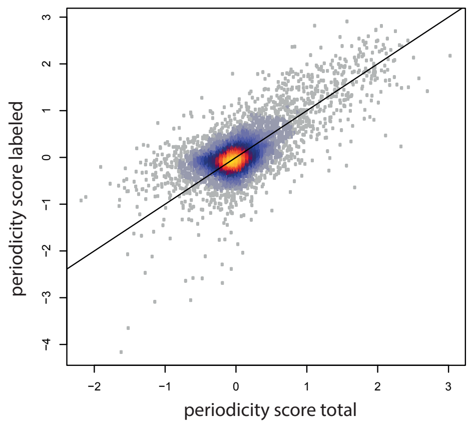

Figure 15: Comparison of individual periodicity scores for total and labeled mRNA. Genes that are presumably non-periodic scatter randomly around the origin, whereas genes having a high score in one of the fractions tend to have also a high score in the other fraction, hence they scatter around the main diagonal.

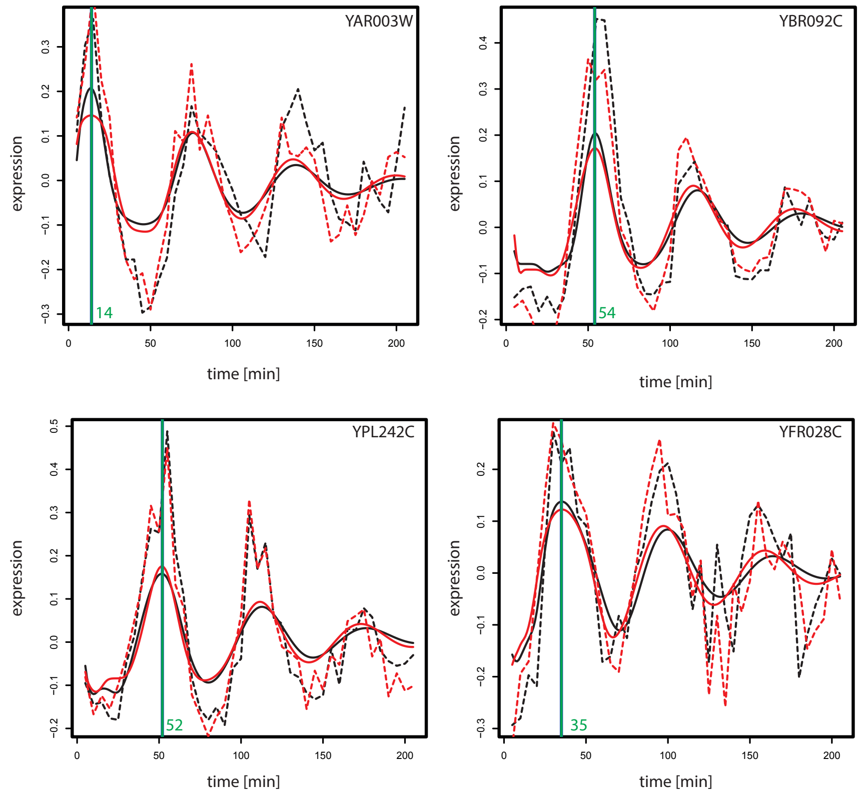

Figure 16: Examples of MoPS fits to total and labeled experimental time courses of selected genes. Total (black) and Labeled (red) time courses (dotted lines) are shown together with the MoPS fitted characteristic time course (solid lines). Here only genes are shown that have the same timing of peak expression in total and labeled (green line). Note that the fitted curves are scaled to L2-norm 1.

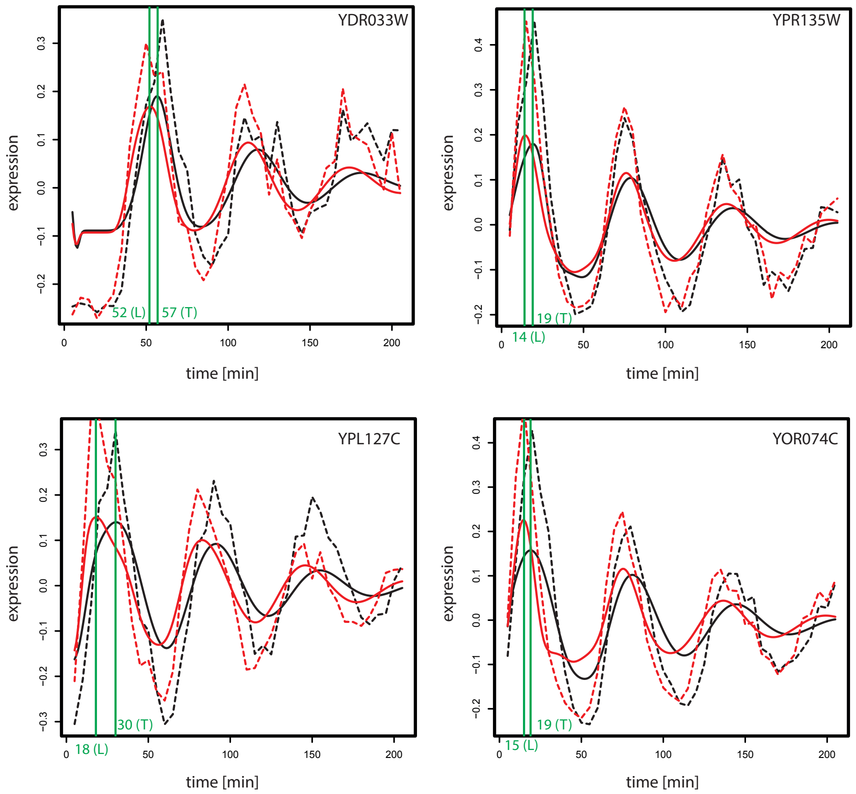

Figure 17: Examples of MoPS fits to total and labeled experimental time courses of selected genes. Total (black) and Labeled (red) time courses (dotted lines) are shown together with the MoPS fitted characteristic time course (solid lines). Shown are genes that show labeled mRNA level peaks that precede total mRNA level as estimated from MoPS (green lines). Note that the fitted curves are scaled to L2-norm 1.

Gene	known phase	estimated phase
CLB6,CLB5,CLN1	late G1	15 min (mean)
CLB3	onset of G2	26 min
Histones	S	29.5 min (mean)
CLB1	onset of M	5.5 min

Table 1: Genes used to assign cell-cycle phases to cDTA cell cycle datasets. Cyclin and histone genes have well defined timing of maximal activity. This information is combined with the MoPS estimated phase of peak total mRNA expression to determine the phases G1,S,G2,M of the cell-cycle.

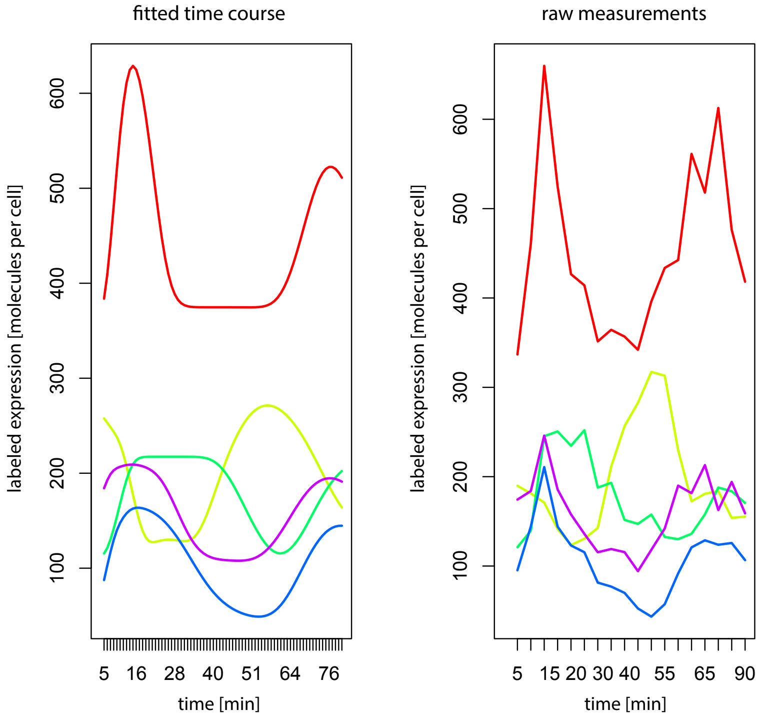

Figure 18: Examples of fitted absolute expression time courses and corresponding raw measurements.

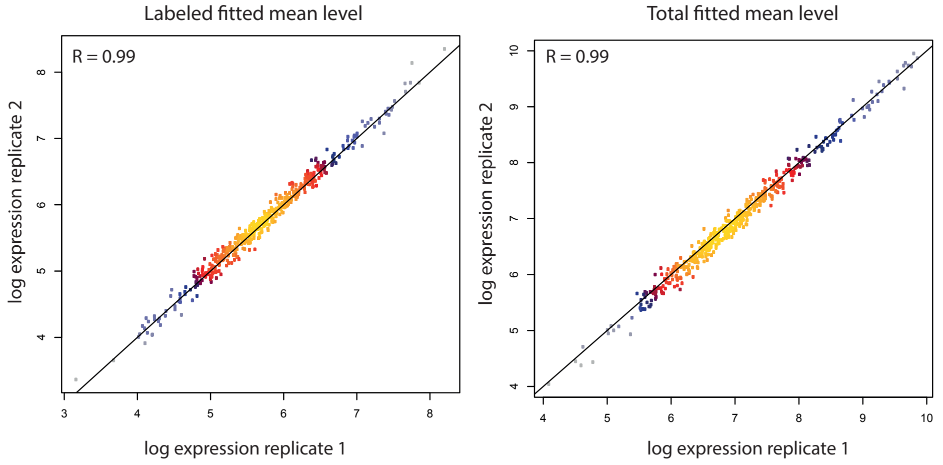

Figure 19: Correlation of estimated absolute mean expression of 479 periodic genes between replicates.

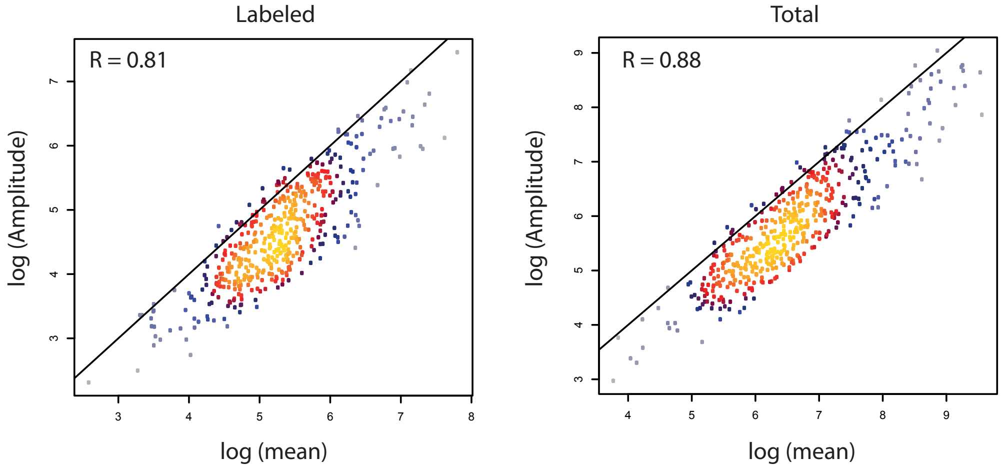

Figure 20: Correlation of mean and amplitude of 479 periodic genes in labeled and total datasets.

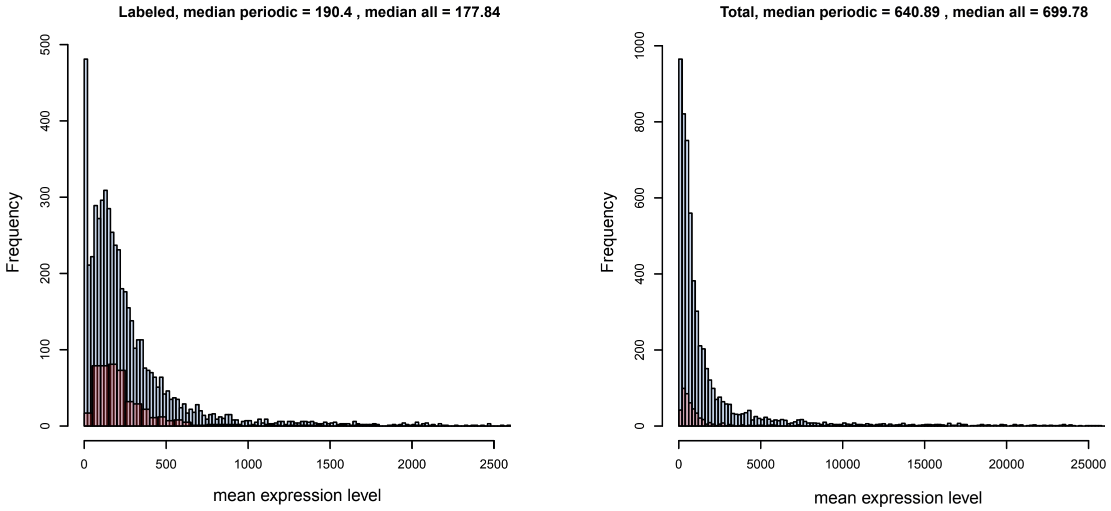

Figure 21: Comparison of mean expression level of 479 periodic (red) and all S.cerevisiae genes (blue) in labeled and total data.

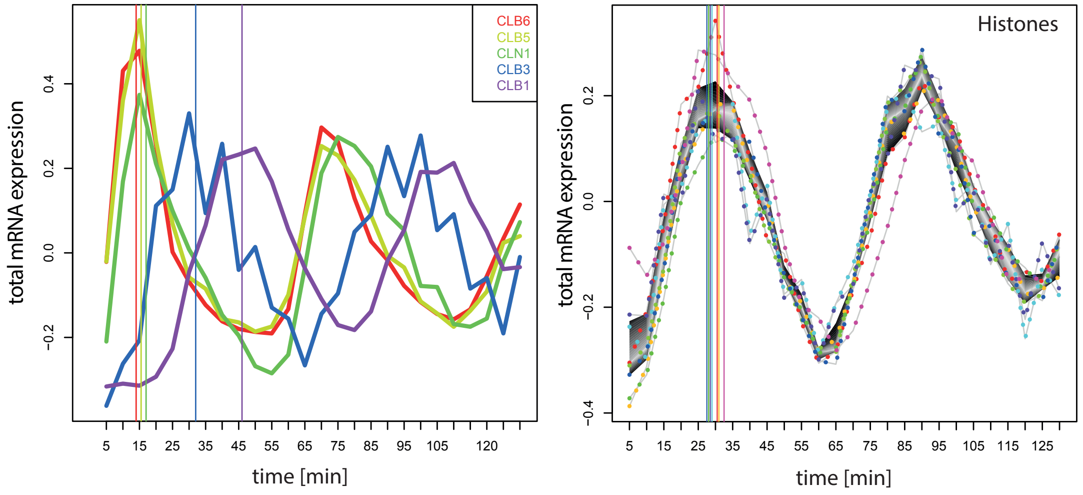

Figure 22: Total mRNA profiles of cyclins and histones. Six cyclins with well-defined cell-cycle stage specific abundance are used to estimate the boundaries of G1,G2 and M phase (left panel). Histone genes (right panel) that show peak timing from 27-32 minutes are used to determine S-phase.

2.8 mRNA synthesis of non-periodic genes during the cell cycle

We investigated the global fluctuation of transcription during the cell cycle. We performed another periodicity screen on labeled data, now using only constant functions as non-periodic test-functions. Among the reliable genes (genes above a certain minimum expression level), we keep the 500 genes with the lowest periodicity scores, i.e., the genes that are most constantly expressed. By averaging and visualizing their normalized (mean expression 1) labeled expression profiles we obtain a lower bound for the global fluctuations in mRNA synthesis (Figure 23). Apart from a global increase in transcription shortly after synchronization, we cannot observe strong global changes in mRNA synthesis.

2.9 Validation of MoPS

2.9.1 Comparison with other cell-cycle expression studies

Despite the existence of many genome-wide cell-cycle expression studies, there is no consensus set of "true" cell-cycle regulated genes. Using different experimental conditions and screening methods 300-1500 cell-cycle genes have been reported in S.cerevisiae. Our set of significantly periodic genes can be compared to other studies by visualising the overlap in identified periodic genes (Figure 24). Three studies are chosen for comparison: Spellman et al. [11] as the pioneering cell-cycle Microarray study; Granovskaia et al. [4] as the most recent study; Cyclebase [3] as a meta-study that combines several studies. Only 246 genes are found to be cell-cycle regulated by all studies, while there are 523 genes that are only identified in one study.

2.9.2 Benchmark on identification of bona-fide cell-cycle genes

We validated our periodicity screening using a framework proposed by de Lichtenberg et al. [2]. They developed their own periodicity screening method and applied it to 6 different cell cycle expression data sets. The resulting 6 ranked lists plus a combined reference list of periodically expressed genes are accessible from the Cyclebase repository [3]. We compared our ranking of periodic genes to these

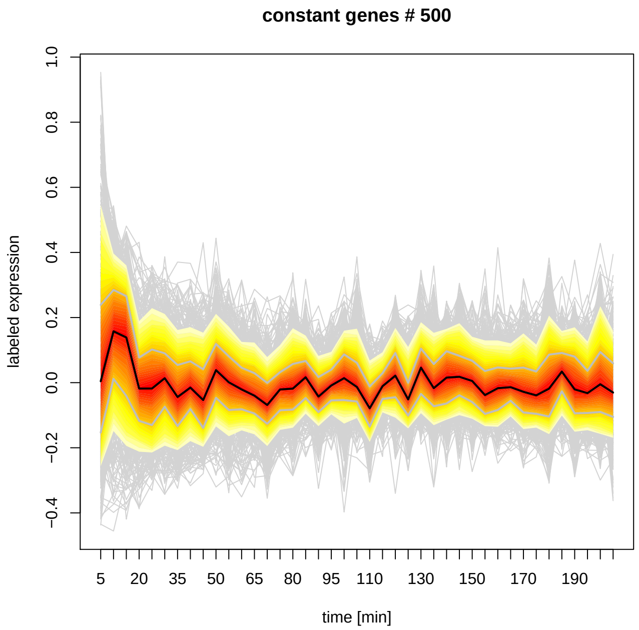

Figure 23: Labeled expression profiles of the 500 most constantly expressed genes. The black line shows the mean value, red and yellow ranges correspond to the 95% and 75% quantiles of the distribution.

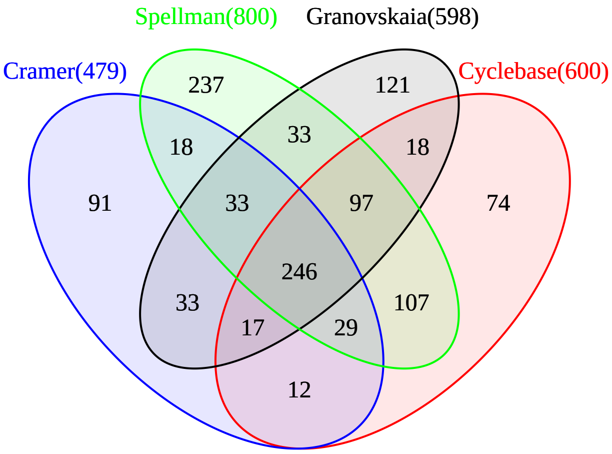

Figure 24: Overlap of MoPS cDTA periodic genes with results from other cell-cycle expression studies.

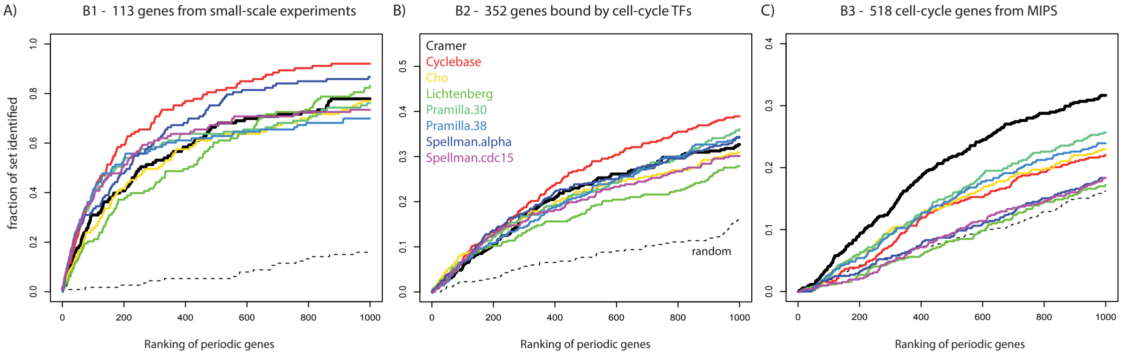

Figure 25: Benchmark of MoPS. Shown are ROC-like curves, one for each ranked list of bona-fide cell-cycle genes. Three different benchmark sets of cell-cycle regulated genes are used as a gold standard for validation. The number of top n genes of a ranked list (x-axis) is plotted versus the fraction of the benchmark set which is contained in the top n genes, respectively. (A) The gold standard set B1 consists of 113 genes identified in small-scale experiments. (B) Benchmark set B2 consists of 352 genes identified in two independent Chromatin IP (ChIP) studies. These genes were found to be bound by known cell-cycle associated transcription factors. (C) Benchmark set B3 consists of 518 genes annotated in MIPS [8] as 'cell cycle' or 'DNA processing'.

7 lists using the benchmark scheme as in [2]. The ranked lists were retrieved from www.cyclebase.org [3]. These lists correspond to different cell-cycle microarray datasets that have been normalized in the same manner and are ranked according to periodicity by the method of Lichtenberg et al. [2]. Additionally, it contains a ranked list that was derived by combining all datasets. Three different benchmark sets were used as a gold standard to assess the quality of a gene list by a receiver operating characteristic (ROC) analysis: set B1 - A total of 113 genes previously identified as periodically expressed in small-scale experiments. Set B2 – 352 genes whose promoters were bound (P-value below 0.01) by at least one of nine known cell cycle transcription factors in two independent Chromatin IP studies. Set B3 – 518 genes annotated in MIPS [8] as 'cell cycle and DNA processing'. A comparison of our ranked list with the other lists was performed as proposed in [2]. In all 7 cases and for all 3 benchmark sets, the de Lichtenberg method has been proven to perform better or at least as good than competing methods [2, 7]. Our ranking, when included in the ROC analysis, performs comparably to the Lichtenberg method in all 3 benchmark scenarios (Figure 25). Out of the top 200 periodic genes from the combined ranking, we find 152 to be significantly periodic with our approach applied to our dataset. Visual inspection shows that the 48 genes that we did not classify as periodic in our dataset, indeed exhibit predominantly periodic profiles in labeled and total but show low correlation between replicates or show deranged profiles in the first 30 minutes after synchronization.

2.9.3 Robustness of peak time assignment

We follow the validation approach as described by Guo et al [5]. to estimate the robustness of peak time assignment to experimental noise. We added varying amounts of gaussian noise to the measured time course of a gene and extract the peak timing of expression. We then compare the perturbed estimates with the original peak times. As in [5], we select the top 100 genes ranked by our periodicity score for benchmarking. For varying levels of noise, we generate 10 perturbed time courses for each gene, estimate the peak time with MoPS and compute the usigned timing differences to the original estimates. The level of noise that is added at every time point is taken from a normal distribution (mean =0, sd = noise.level * error). The error is estimated from the calculated variation in our

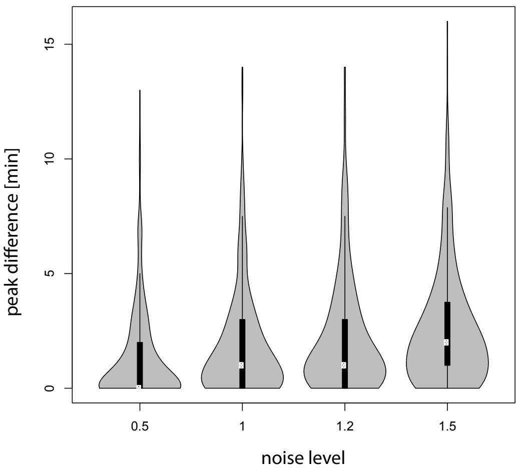

Figure 26: Violin plot showing peak time variation in simulated perturbed expression measurements. Each violin shows the distribution of unsigned differences between peak timing estimated from original and perturbed time courses (labeled data).

experimental replicate time series (see Section 1.2). We use four different levels of noise: 0.5 (more precise than actual measurements), 1, 1.2 and 1.5. The median peak time deviation was in the range of 1-2.5 min, confirming the accuracy of our wobblet estimates.

2.10 Regulation of periodic mRNA synthesis timing by TFs

We used XXmotif and TOMTOM together with ChIP-chip derived TF - target associations to identify TFs that regulate cell-cycle regulated mRNA expression (see Methods, main text). A total of 32 TFs have been found with this approach. The complete list and information on the number of associated periodic genes is given in the Supplementary Materials.

We investigated the labeled time courses of cell-cycle genes that are regulated by the same TF(s). Several combinations of TFs are found in our analyses that are specific to a subset of our 479 periodic genes. The labeled expression profiles are highly similar within these subsets and show a coherent timing of peak expression (Figure 29).

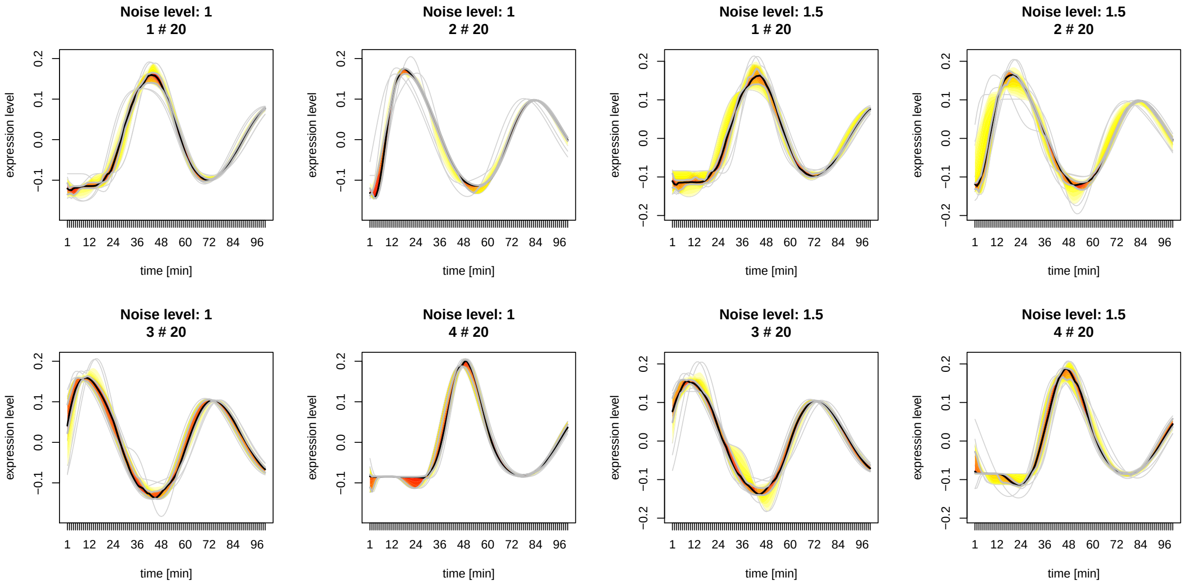

Figure 27: Clusterplots of MoPS time courses fitted to 20 randomly perturbed expression profiles of four selected genes. Shown are fitted time courses estimated from expression measurements that have been perturbed with noise level 1 (left panel) and 1.5 (right panel).

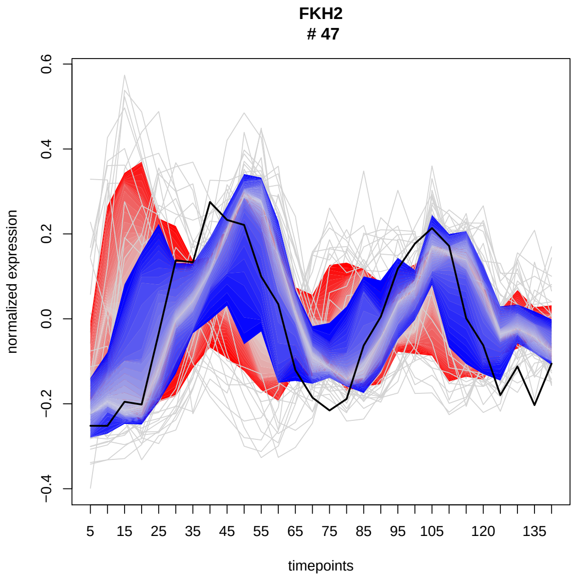

Figure 28: Labeled expression time courses of 47 targets of the cell-cycle transcription factor FKH2. FKH2 total mRNA peaks at the beginning of M-phase (black line). One group of periodic targets show labeled expression peaks approx. 10 minutes after FKH2 peak expression (blue), a second group comprises genes that show labeled peak expression when FKH2 levels are low (red).

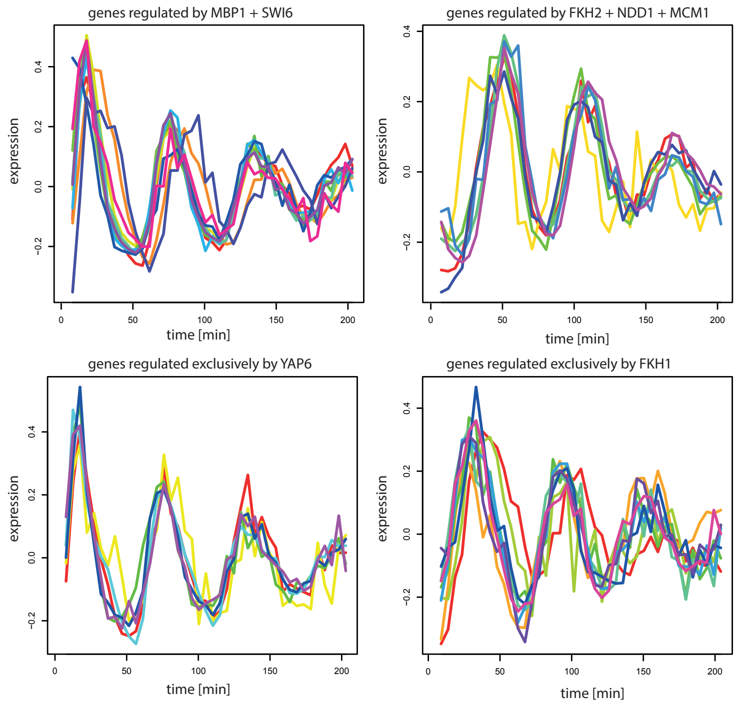

Figure 29: Periodic genes that are regulated by common TF(s). Each panel shows labeled expression time-courses of genes that are regulated by a common set of TFs. The lower two panels show genes that are regulated by only one cell cycle TF.

3 Dynamic RNA turnover model and screen for periodic fluctuations in RNA degradation

3.1 A model for mRNA synthesis and degradation

Let $T(t)$ respectively $L(t)$ denote the time-dependent total respectively labeled mRNA amount of a certain transcript at time t. We assume that the mRNA population of a gene is synthesized with a time-dependent synthesis rate $\mu = \mu(t)$. We further assume that mRNA decays exponentially at a time-dependent rate $\delta = \delta(t)$. The amount of degraded mRNA molecules during the time interval dt can be expressed as $\delta(t)T(t)$. The synthesis rate function $\mu(t)$, the decay rate function $\delta(t)$ and the initial mRNA level $T(0)$ determine the total expression $T(t)$ and its labeled expression $L(t)$ by the differential equation

$$\frac{dT(t)}{dt} = \mu(t) - \delta(t)T(t). \tag{25}$$

Note that in [9], [12], we needed to account for an increase in the cell number with time. Here, we only follow the first cell cycle of the experiment, because the fluctuations in later cell cycles are attenuated too much to be informative for degradation estimation. Without loss, we may therefore assume a constant cell number, which simplifies our calculations considerably. Furthermore, we do not model cell growth, since we follow a synchronized population of cells for one cell cycle, therefore the growth rate $\alpha = 0$. Equation (25) can be solved efficiently for arbitrary, sufficiently smooth functions μ and δ using a numerical ODE solver. Assuming piecewise linear functions for μ and piecewise constant functions for δ, it is even possible to derive the analytical solution to Equation (25).

We start labeling at time point t_0 and set

$$\Theta_g(t, t_0) := \int_{t_0}^{t} \delta(\xi) d\xi \tag{26}$$

The slope of the piecewise linear function for μ changes at time points m_i with $i = 0, ..., k$. The piecewise constant degradation rate δ changes at d_i with $i = 0, ..., n$. We set $H := \{h_i \mid i = 0, ..., k+n\} = \{m_i \mid i = 0, ..., k\} \bigcup \{d_i \mid i = 0, ..., n\}$with $h_i \leq h_{i+1}$ for all i. On each interval $[a, b]$ $(a = h_i, b = h_{i+1})$ we can calculate

$$\phi(a, b) = \int_a^b \left[\mu^a + \frac{\mu^b - \mu^a}{m^b - m^a} \cdot (\xi - m^a)\right] e^{\alpha\xi + \Theta(\xi, 0)} d\xi\,.$$

Equation (25) can then be solved as

$$T(t) - T(t_j) = e^{-\Theta(t, t_j)} \left[T(t_j) + N \sum_{i|t>h_i} \phi(h_{i-1}, h_i) + \phi(h_{max}, t)\right] \tag{27}$$

with $h_{max} = max(h_i > t)$. The total amount of mRNA can therefore be derived using $t_j = 0$ and $T(0) = T_0$. The amount of labeled mRNA at time point t_j is obtained from Equation (27) by $L(t_j) = T(t_j + t_{lab}) - T(t_j)$, where t_{lab} is the length of the labeling interval.

3.2 Model specification

Given total and labeled time courses $T(t_k, i)$ and $L(t_k, i)$ of a gene in replicates $i \in I$, our main purpose is testing for the existence of periodic changes in mRNA degradation. We compare a model with constant decay rate, $\delta(t) = \delta$, with a model for regulated decay, in which $\delta(t)$ is a cosine function with average decay level δ_m, peak time φ and amplitude a. The synthesis rate $\mu(t)$ is modeled as a piecewise linear function with 10 min intervals between interpolation points (5 min, μ_0), (15 min, μ_1), ..., (65 min, μ_6). Additionally, we need to rescale the measured labeled mRNA fractions $L(t)$ by an unknown factor c in order to match the true fraction of newly synthesized mRNA given the amount of measured total mRNA. This parameter reflects the true ratio between the (mean) labeled expression measurements and the (mean) total expression measurements of all genes at time 0. Given a complete parameter set Θ for one of the models, the snynthesis and degradation rates are then converted into predctions for the labeled and total mRNA time corses, $\hat{T}(t_k; \Theta)$ and $\hat{L}(t_k; \Theta)$. Our target $l(\Theta)$ function measures the goodness-of-fit for both time courses, where goodness-of-fit is given by Equation (3). Hence,

$$\begin{aligned} \ell(\Theta) &= \sum_{i \in I} \ell(\Theta; T(t_k, i)) + \sum_{i \in I} \ell(\Theta; L(t_k, i)) \\ &= \sum_{i \in I} \sum_{k=1}^{K} \frac{(log\, T(t_k, i) - log\, \hat{T}(t_k; \Theta))^2}{2 \cdot \sigma^2_{T,t_k,i}} + \sum_{i \in I} \sum_{k=1}^{K} \frac{(log\, L(t_k) - \frac{1}{c} \cdot log\, \hat{L}(t_k; \Theta))^2}{2 \cdot \sigma^2_{L,t_k,i}} \end{aligned} \quad (28)$$

where $\sigma^2_{T,t_k,i}$ and $\sigma^2_{L,t_k,i}$ are the regularized replicate-, gene- and time-specific standard deviations obtained in Section 1.2.

Thus, the full model M_1 assuming constant decay for one gene is parametrized by

$$\Theta_{M_1} = \{c,\, \delta,\, \mu_0, ...,\, \mu_k\} \quad (29)$$

and the competing model M_2 using a sigmoidal function for the decay is parametrized by

$$\Theta_{M_2} = \{c,\, \delta_m,\, \varphi,\, a,\, \mu_0, ...,\, \mu_k\} \quad (30)$$

Both models are fitted using standard Metropolis-Hastings MCMC (we use Gaussian proposal functions truncated to the positive real values).

3.3 Detection of genes with variable degradation rate

Applying both the constant and the regulated decay model to a gene profile, this results in a score for the constant model Θ_{M_1} and a score for the regulated Model Θ_{M_2} (compare Equation (28)). For each gene profile we compare the fit of the two models by calculating a *Variable Degradation Score,* VDS, which is given by the log-likelihoodratio ratio between the two models:

$$VDS = \ell(\Theta_{M_1}) - \ell(\Theta_{M_2}) \quad (31)$$

Since constant degradation is a special case of variable degradation with a max/min ratio of 1, the constant degradation model never scores better than the variable degradation model. Consequently, the Variable Degradation Score is never negative. It is zero when both models fit equally well, and it is higher the more the variable degradation model is required to explain the data. By simulations we determined the sensitivity and specificity of different Variable Degradation Score cutoffs (main text, Figure 6D). We concluded that a Variable Degradation Score cutoff of 0.3 ensures sufficiently high sensitivity and specificity for genes with a degradation rate amplitude (max/min ratio) of at least 1.5.

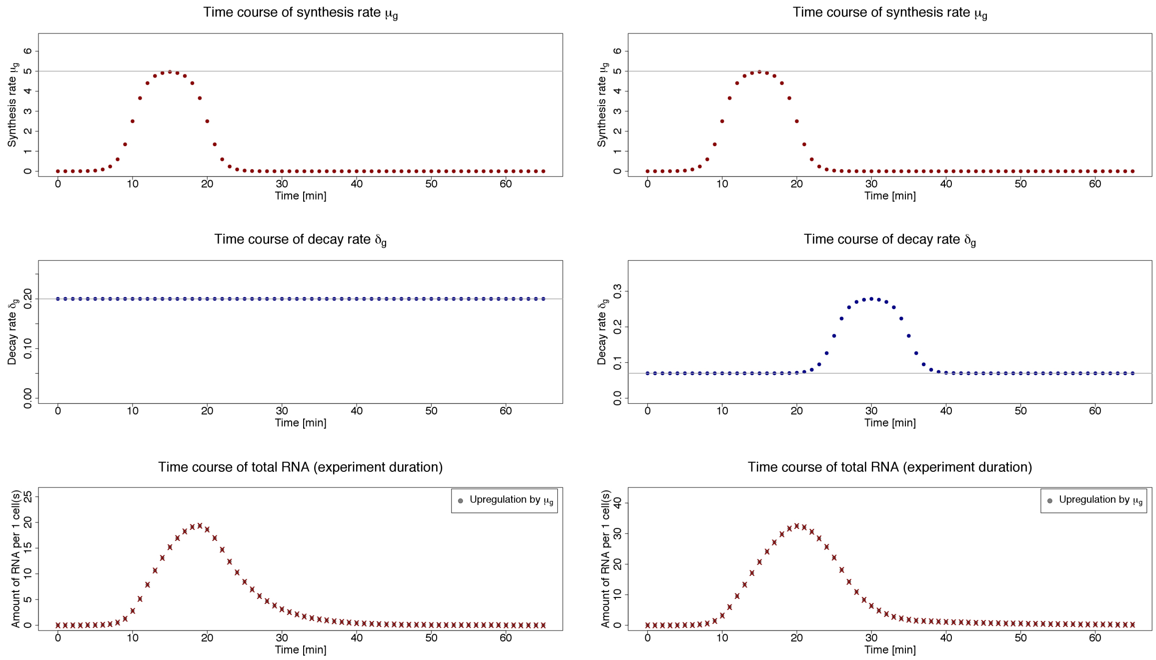

Figure 30: Comparison of total mRNA time courses achieved by a high, constant degradation rate (left) and a variable, peaked degradation rate (right). The top panel shows the synthesis rate, which is identical in both scenarios. The middle panel shows the degradation rate, while the lower panel shows the total mRNA time course resulting from the given synthesis and decay rates. Though the total mRNA profiles have a similar shape, peak total mRNA levels are more than 50% higher in the variable degradation scenario.

3.4 Sensitivity and specificity of the Variable Degradation Score in simulated data

Our main task is to distinguish profiles of periodically expressed genes with constant degradation from those with a fluctuating degradation rate. This task becomes increasingly difficult with a higher basal degradation rate, because changes in mRNA synthesis are immediately mirrored in total mRNA levels.

Gene profiles with a sharply peaking synthesis rate are likely to look very similar when either a high constant mRNA degradation is assumed or a variable degradation rate peaking shortly after the synthesis rate (Figure 30).

The sensitivity of the Variable Degradation Score was assessed in a simulation study. The simulated data contained gene profiles with either constant or variable degradation rates while having identical, variable synthesis rates. The synthesis rate time courses were constructed from a sigmoidal curve rising from 0 transcripts/min to a level between 2 and 20 transcripts/min within a 10min time interval. Similarly, after reaching its maximum, the synthesis rate curve returned to 0 transcripts/min in a sigmoidal curve. 10 constant degradation rates were chosen in the range from $0.1\,\text{min}^{-1}$ to $0.5\,\text{min}^{-1}$. The variable degradation rates had the same shape as the synthesis rates, but their peak was delayed 15min after the respective synthesis rate peak. The max/min degradation ratio was set to 4, with a mean degradation rate in the same range as the constant degradation rates. With these artificial synthesis and degradation rate curves, we calculated idealized labeled and total measurements.

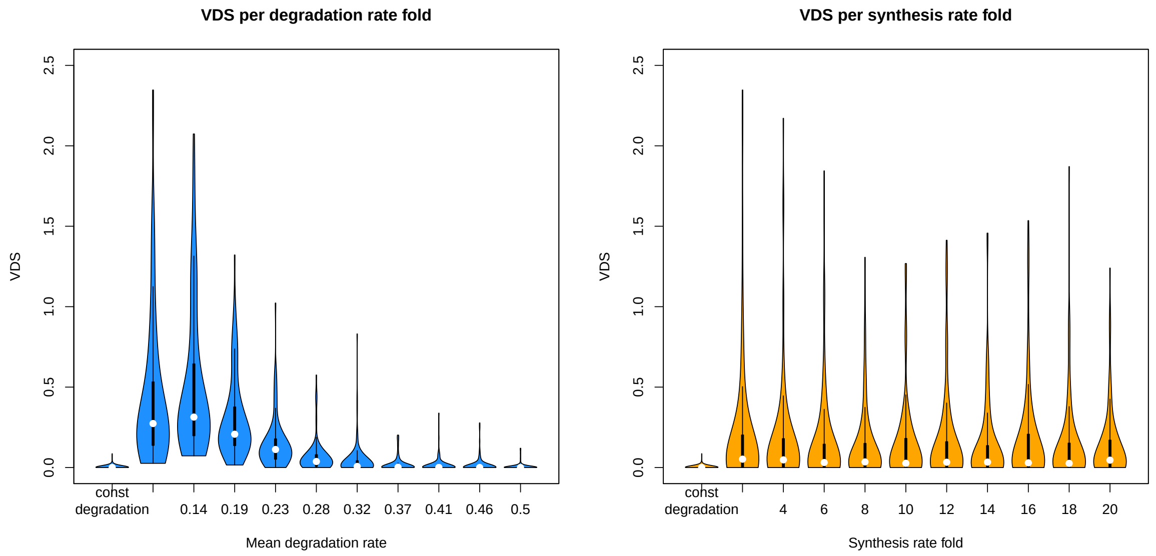

Figure 31: The Variable Degradation Score depends on the magnitude of the degradation rate, but not on the amplitude of the synthesis rate fluctuations. The VDSs were calcualted for a comprehensive set of artificial genes (see paragraph 3.4). Left: The VDSs of genes with fluctuating degradation rates are grouped according to their mean (true) degradation rate, and their respective distribution is shown as a violin plot. For comparison, the lefmost violin represents the VDS distribution of the constant genes. Among those genes with a fluctuating degradation rate, the average VDS decreases with the mean degradation rate. Right: The VDSs of genes with fluctuating degradation rates are grouped according to the amplitude of the synthesis rate fluctuations. For comparison, the lefmost violin represents the VDS distribution of the constant genes. The VDS does not depend visibly on the synthesis rate fold.

Most importantly, the results revealed that the Variable Degradation Score has sufficient power for average degradation levels up to 0.3. This corresponds to a half-life of about 2.3min (Figure 31). Assuming higher degradation rates, periodic profiles can be explained sufficiently well by adaptation of the synthesis rate only. The constant degradation model does not fit significantly worse than the variable degradation model in this case. On the other hand, the sensitivity of the Variable Degradation Score does not depend on the fold of the synthesis rate fluctuations (Figure 31).

3.5 The time shift of degradation vs synthesis determines the efficiency of regulation

To examine the influence of the time delay between synthesis and degradation peak time on the amplitude of total mRNA expression, we conducted a simulation. Therefore, the same cosine-shaped synthesis rate was used while shifting the cosine-shaped degradation rate from 0 to 2π $(= cellcycle\, length = 60min)$. The corresponding total mRNA levels were computed according to Equation (27) and are shown in Figure 32. Due to the periodicity property of our model, the degradation rate peak shift by 2π corresponds to the results where the degradation rate is not shifted, and thus results for the shift by 2π are not shown here.

The amplitudes of the total level vary with the shift of the degradation rate from the synthesis rate. The maximal amplitude in the total level is reached when the degradation rate is shifted by $\pi = 30min$.

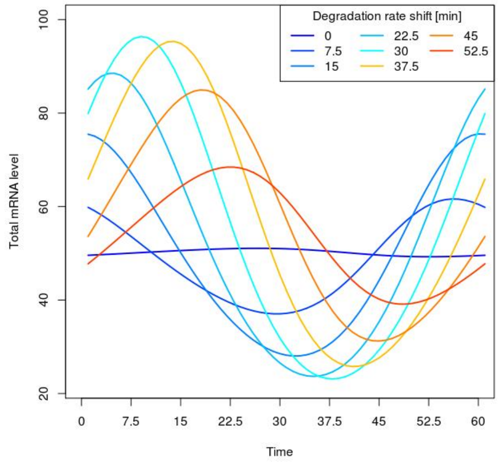

Figure 32: Total mRNA time courses retrieved using degradation rates that only differ in their degradation peak shift relative to the synthesis rate peak. The resulting amplitudes of the total mRNA levels vary.

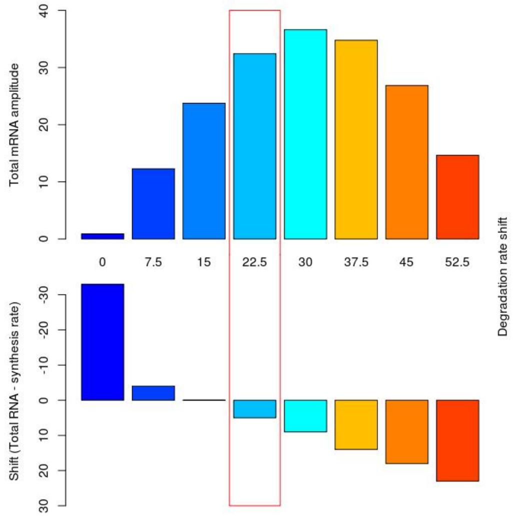

Figure 33: Shifting the cosine shaped degradation rate relative to the synthesis rate results in different amplitudes and peak times of the total mRNA level. Top: Derived amplitudes for the total mRNA expression level. Bottom: Shift between the peak times of the total mRNA levels and the synthesis rate. The red rectangle indicates the bars which correspond to the degradation rate shift which is most similiar to the one we observed for our set of regulated periodic genes (21min).

Figure 33 shows the resulting amplitudes and peak time delays between the total RNA level and the synthesis rate for the simulated shifts in degradation rate peak time. For our set of periodic genes with variable degradation the observed peak time delay between synthesis and degradation rate corresponds to 21 min. Figure 33 indicates that this delay achieves a good balance between a short peak delay between the total mRNA and the synthesis rate, while the amplitude of the expression level is relatively high. This suggests that the time delay between the synthesis rate peak and the degradation rate peak is crucial for effiecient transcription regulation.

4 Modeling of mRNA synthesis and degradation in cDTA cell cycle data

Both degradation models were applied to cDTA measurements from S. cerevisiae and time-dependent synthesis and degradation rates were estimated for each gene. The estimated parameters for Θ_{M_2}(Section 3.2) are listed in Supplemental Tables 3 and 4. Figure 34 shows the fitting results for a gene where constant mRNA degradation is very likely, as both models resulted in almost the same loss and yielded similiar results. This indicates, that the regulated model, which was started with the parameter set the constant model returned, could not improve the fitting accuracy. Furthermore, it can be seen, that for this gene, the derived degradation rates are both very similar to the wild-type degradation rate from [12].

Figure 35 shows fitting results for a periodically expressed gene, *Ace2,* where a regulated mRNA degradation rate is more likely and explains the measured data better. The fitting results show that due to the sine-shaped degradation rate the synthesis rate in the regulated model is also adapted and differs from the synthesis rate in the constant model. The degradation rate from the constant model is higher than the mean variable degradation rate, which is probably due to the fact that high degradation rates allow to explain mRNA levels only by synthesis rates.

4.1 Improvement of variable degradation model over constant degradation model

The variable degradation model enables us to explain sharp peaks in total mRNA better than a constant degradation rate and is thus an improvement over the constant mRNA degradation, as it results in lower loss values and higher log-likelihood-ratios, respectively. Figure 36 shows for corresponding constant loss values, how much the variable degradation model improves the fitting results. For small losses yielded by the constant model the variable degradation model proposes almost no improvement. But the higher the loss in the constant model, which is achieved due to a bad fitting process, the higher is the reduction in the loss of the variable degradation model compared to the constant degradation model. The sine-shaped degradation rate seems to provide a valuable tool to improve the fitting results and therefore helps to explain those measured gene profiles better.

4.2 Transcription regulation by degradation rate peaks subsequent to synthesis rate peaks

As described in Section 3.5 the time shift between the synthesis rate peak and the degradation rate peak is crucial for the regulation of mRNA expression. For those periodically expressed genes predicted to have a variable degradation rate we compared the peak times of their synthesis and degradation rates. Although highly correlated, we see a clear delay of the degradation rate peak times compared to the synthesis peaks of about 21 minutes (Figure 37). Furthermore we used the cell cycle boundaries

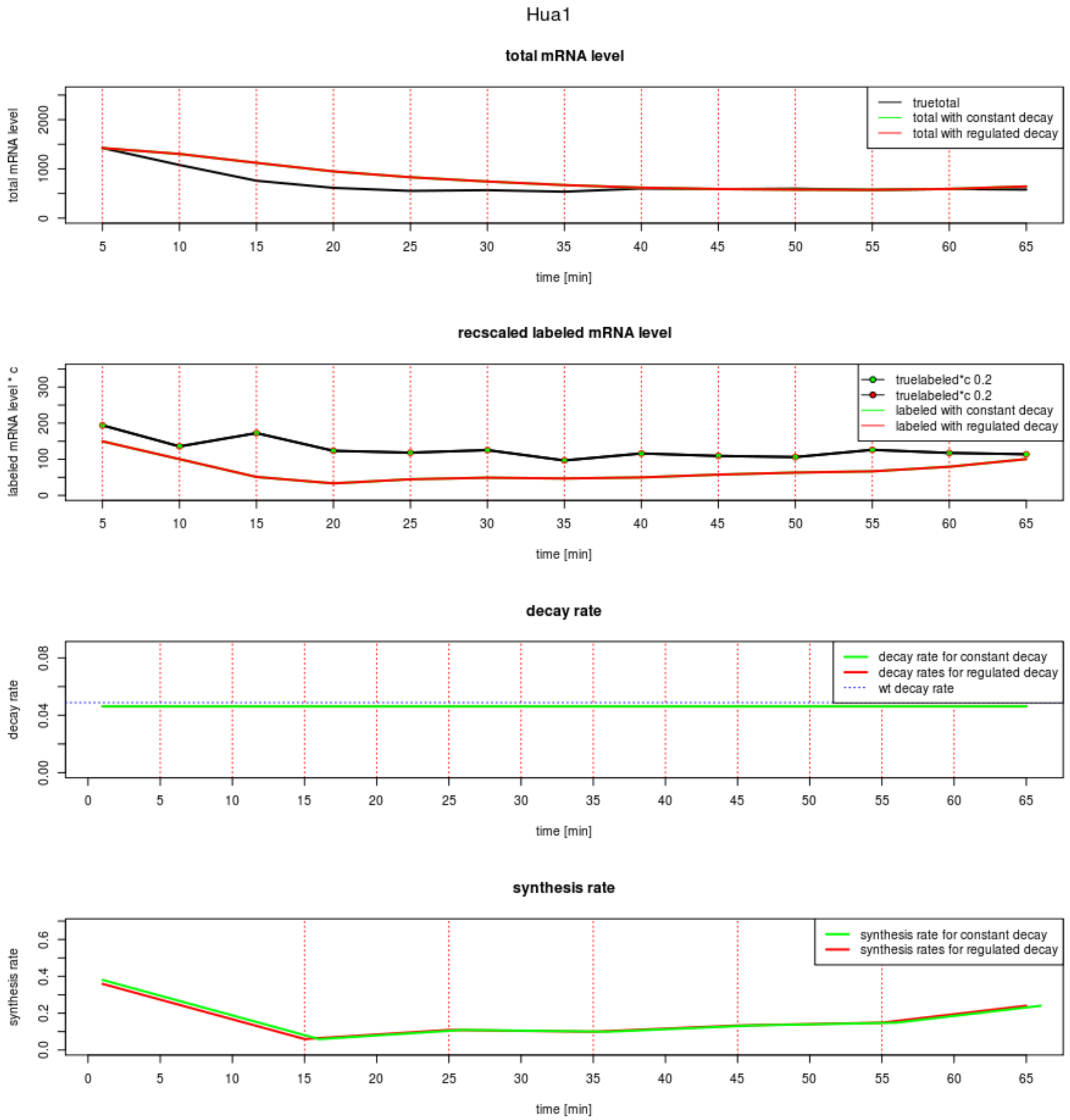

Figure 34: Fitting results for the *Hua1* gene for both the constant and the regulated model using a piecewise linear synthesis rate. The upper panel shows the measured total mRNA level (black) together with the estimated total levels from the constant (green) and regulated (red) degradation model. The second panel shows measured and estimated labeled time courses, color coded as above. Estimated labeled mRNA abundances are multiplied by $c = 0.2$. The third and the fourth panel show the estimated decay and synthesis rates for the constant (green) and regulated (red) mRNA degradation model. Additionally, in panel 3 the wild-type degradation rate from [12] is highlighted as a dotted blue line.

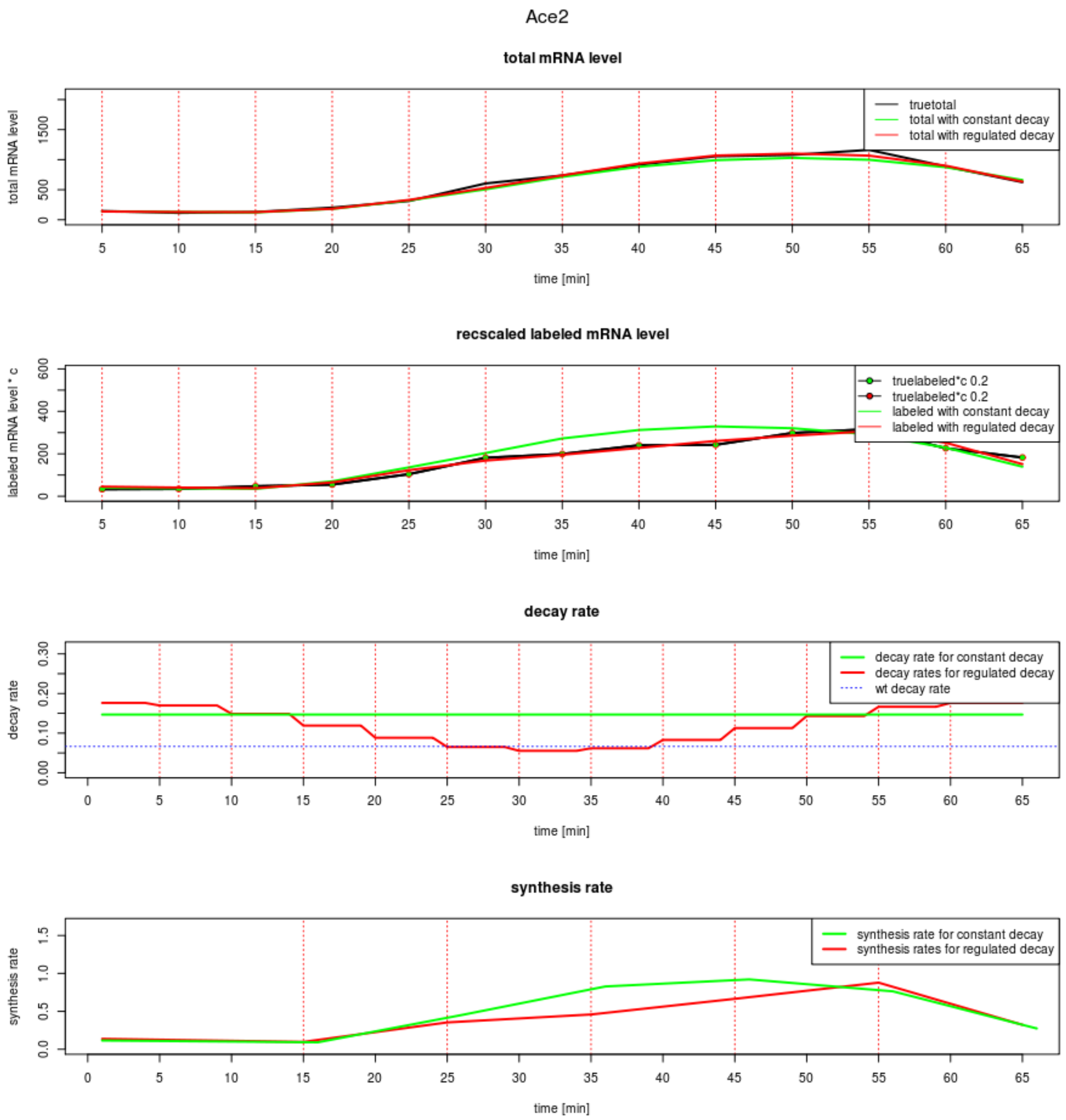

Figure 35: Fitting results for the *Ace2* gene. The upper panel shows the measured total mRNA level (black) together with the estimated total levels from the constant (green) and regulated (red) degradation model. The second panel shows measured and estimated labeled time courses, color coded as above. The third and the fourth panel show the estimated decay and synthesis rates for the constant (green) and regulated (red) mRNA degradation model. Additionally, in panel 3 the wild-type degradation rate from [12] is highlighted as a dotted blue line.

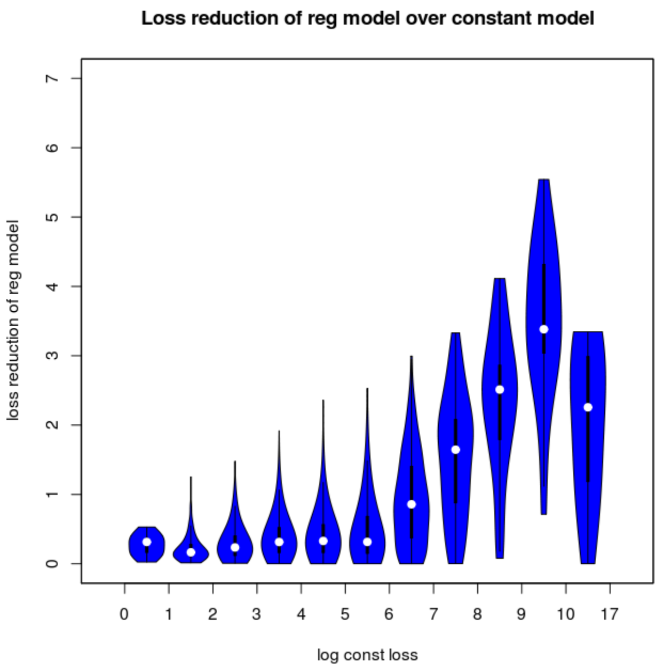

Figure 36: Loss reduction of variable degradation model over constant degradation model. Logarithmic constant losses were binned. The violins show the distribution of frequencies of the loss reduction in the variable degradation model over the constant degradation model for genes whose constant loss lies in the corresponding bin. White marks on the violin plots indicate the median value for the loss reduction, the black lines mark the 25/75% inter-quartile range.

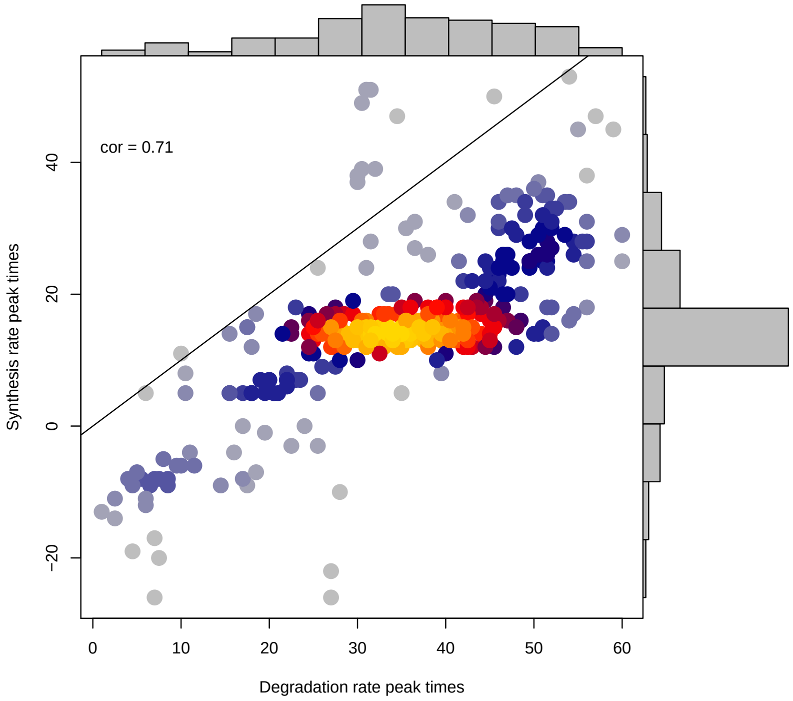

Figure 37: Correlation of degradation rate peak times (x-axis) and synthesis rate peak times (y-axis) for periodically expressed genes with regulated degradation. For genes where the synthesis peaks at the end of a cell cycle and the degradation rate peaks at the beginning of the subsequent cell cycle, the synthesis rates were shifted by -60 minutes. The spearman correlation is 0.71.

as derived from cyclin and histone peak (Section 2.6) and gouped the genes according to the cell cycle phases in which their synthesis peak times fall into. The degradation rates of the genes in the corresponding groups peak shortly after the respective cell cycle phases of the synthesis rates (main Figure 6E).

4.3 Correlation between the periodicity score and the variable degradation score

As described in the main text, the variable degradation score and the periodicity score are positively correlated. Figure 38 shows the variable degradation scores (VDS) for genes which are subject to periodic transcription. The increasing VDS with increasing periodicity leads us to the conclusion that periodic fluctuations in the mRNA degradations are coupled to periodic transcription.

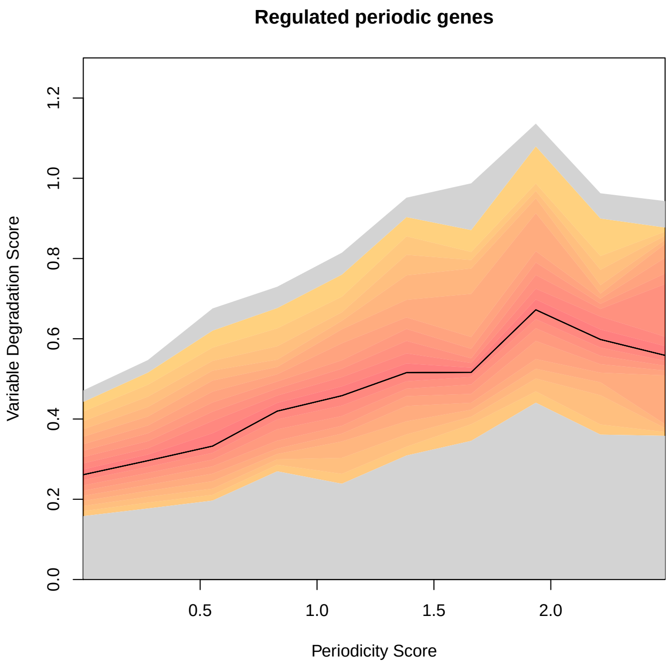

Figure 38: Correlation between the periodicity score and the variable degradation score for 2584 genes with periodicity score > 0. Quartiles of the regulated-degradation score distribution (y-axis) are shown as a function of the periodicity score (x-axis). The interquartile range (25%-75% quantile is shown in orange-red, the extreme regions (0%-25% and 75%-100% quantile) are shown in grey, the central black line is the median line

5 Supplementary Tables

Supplementary Table 1

Identified DNA motifs in clusters of co-expressed cell cycle genes in the region 500bp upstream of the TSS. Transcription factors that are significantly associated with identified motifs. The column "TF periodic" indicates if the TF is periodically expressed. The column "number of periodic targets" shows the number of target genes that are periodically expressed.

Available for download as XLSX file.

Supplementary Table 2

Table of all fitting results for the periodic degradation rates and the corrsponding synthesis rates of all transcripts for replicate 1. Transcript column: Systematic names for Saccharomyces cerevisiae transcripts. Score column: Variable Degradation Score (VDS) for the corresponding transcript with the best fitting synthesis and degradation rates. Delta column: Average decay level round which the periodic degradation rate oscillates. Phi column: Peak time point of periodic, cosine-shaped degradation rate with wave length equals cell cycle time. Mu columns: Piecewise linear modeled synthesis rates in molecules per minute per cell at the respective time points of 5,15,25,35,45,55, and 65 minutes after release of cells in G1 phase of the cell cycle.

Available for download as XLSX file.

Supplementary Table 3

Table of all fitting results for the periodic degradation rates and the corrsponding synthesis rates of all transcripts for replicate 2. Transcript column: Systematic names for Saccharomyces cerevisiae transcripts. Score column: Variable Degradation Score (VDS) for the corresponding transcript with the best fitting synthesis and degradation rates. Delta column: Average decay level round which the periodic degradation rate oscillates. Phi column: Peak time point of periodic, cosine-shaped degradation rate with wave length equals cell cycle time. Mu columns: Piecewise linear modeled synthesis rates in molecules per minute per cell at the respective time points of 5,15,25,35,45,55, and 65 minutes after release of cells in G1 phase of the cell cycle.

Available for download as XLSX file.

Supplementary Table 4

cDTA synthesis rate estimates of all S.cerevisiae genes. The 82 labeled mRNA samples from both time series are treated as replicates. The columns "median", "sd", "mean", "cv", "95%-confidence" correspond to the median, standard deviation, mean, coefficient of variation and 95% confidence interval of the distribution of estimated synthesis rates, respectively.

Available for download as XLSX file.

Supplementary Table 5

cDTA half-life estimates of all S.cerevisiae genes. The 82 total and 82 labeled mRNA samples from both time series are treated as replicates and are used to determine the half-lifes of all mRNAs. The columns “median”, “sd”, ”mean”, ”cv”, ”95%-confidence” correspond to the median, standard deviation, mean, coefficient of variation and 95% confidence interval of the distribution of estimated half-lifes, respectively.

Available for download as XLSX file.

References

[1] W. S. Cleveland. Robust locally weighted regression and smoothing scatterplots. *Journal of the American Statistical Association*, 74(368):829–836, 1979.

[2] U. de Lichtenberg, R. Wernersson, T. S. Jensen, H. B. Nielsen, A. Fausbøll, P. Schmidt, F. B. Hansen, S. Knudsen, and S. Brunak. New weakly expressed cell cycle regulated genes in yeast. *Yeast*, 22(15):1191–1201, 2005.

[3] N. P. Gauthier, M. E. Larsen, R. Wernersson, U. de Lichtenberg, L. J. Jensen, S. Brunak, and T. S. Jensen. Cyclebase.org - a comprehensive multi-organism online database of cell-cycle experiments. *Nucleic Acids Research*, 36(Database issue):D854–D859, 2008.

[4] M. V. Granovskaia, L. J. Jensen, M. E. Ritchie, J. Toedling, Y. Ning, P. Bork, W. Huber, and L. M. Steinmetz. High-resolution transcription atlas of the mitotic cell cycle in budding yeast. *Genome Biology*, 11(3):R24–R24, 2010.

[5] X. Guo, A. Bernard, D. A. Orlando, S. B. Haase, and A. J. Hartemink. Branching process deconvolution algorithm reveals a detailed cell-cycle transcription program. *Proceedings of the National Academy of Sciences*, 110(10):E968–E977, 2013.

[6] X. Lu, W. Zhang, Z. S. Qin, K. E. Kwast, and J. S. Liu. Statistical resynchronization and Bayesian detection of periodically expressed genes. *Nucleic Acids Research*, 32(2):447 –455, 2004.

[7] S. Marguerat, T. S. Jensen, U. de Lichtenberg, B. T. Wilhelm, L. J. Jensen, and J. Bähler. The more the merrier: comparative analysis of microarray studies on cell cycle-regulated genes in fission yeast. *Yeast*, 23(4):261–277, 2006.

[8] H.-W. Mewes, D. Frishman, U. Güldener, G. Mannhaupt, K. Mayer, M. Mokrejs, B. Morgenstern, M. Münsterkötter, S. Rudd, and B. Weil. Mips: a database for genomes and protein sequences. *Nucleic acids research*, 30(1):31–34, 2002.

[9] C. Miller, B. Schwalb, K. Maier, D. Schulz, S. Dümcke, B. Zacher, A. Mayer, J. Sydow, L. Marcinowski, L. Dölken, D. E. Martin, A. Tresch, and P. Cramer. Dynamic transcriptome analysis measures rates of mRNA synthesis and decay in yeast. *Mol Syst Biol*, 7, 2011.

[10] T. Pramila, W. Wu, W. Stafford Noble, and L. Breeden. Periodic genes of the yeast Saccharomyces cerevisiae: A combined analysis of five cell cycle data sets. `http://labs.fhcrc.org/breeden/cellcycle/index.html`.

[11] P. T. Spellman, G. Sherlock, M. Q. Zhang, V. R. Iyer, K. Anders, M. B. Eisen, P. O. Brown, D. Botstein, and B. Futcher. Comprehensive Identification of Cell Cycle-regulated Genes of the Yeast Saccharomyces cerevisiae by Microarray Hybridization. *Molecular Biology of the cell*, 9(12):3273–3297, 1998.

[12] M. Sun, B. Schwalb, D. Schulz, N. Pirkl, S. Etzold, L. Larivière, K. C. Maier, M. Seizl, A. Tresch, and P. Cramer. Comparative dynamic transcriptome analysis (cDTA) reveals mutual feedback between mRNA synthesis and degradation. *Genome Research*, 22(7):1350–1359, 2012.

[13] D. Zenklusen, D. R. Larson, and R. H. Singer. Single-RNA counting reveals alternative modes of gene expression in yeast. *Nature Structural & Molecular Biology*, 15:1263–1271, 2008.
